# Mitochondria-derived peptide hydrogel augments mitochondrial transplantation for promoting cardiac repair via macrophage metabolic reprogramming

**DOI:** 10.1016/j.bioactmat.2026.06.010

**Published:** 2026-08-14

**Authors:** Hang Li, Yushan Zhang, Yifan Jian, Fang Fang, Wenbin Ouyang, Donglin Zhuang, Wenhao Ju, Rui Gao, Yu Gao, Shaoyang Kang, Pengxu Kong, Yuwei Li, Xiangbin Pan, Weiwei Wang, Zujian Feng

**Affiliations:** a Department of Structural Heart Disease, Fuwai Hospital & National Center for Cardiovascular Disease, State Key Laboratory of Cardiovascular Disease, Chinese Academy of Medical Sciences & Peking Union Medical College, National Clinical Research Center for Cardiovascular Diseases, Beijing, 100037, China; b State Key Laboratory of Advanced Medical Materials and Devices, Institute of Biomedical Engineering, Chinese Academy of Medical Science and Peking Union Medical College, Tianjin, 300192, China; c State Key Laboratory of Medicinal Chemical Biology, Key Laboratory of Bioactive Materials (Ministry of Education), College of Life Sciences, Nankai University, Tianjin, 300350, China; d Department of Thoracic Surgery, The First Affiliated Hospital, School of Medicine, Zhejiang University, Hangzhou, 310000, China; e Department of Structural Heart Disease, Fuwai Shenzhen Hospital, Chinese Academy of Medical Sciences, Shenzhen Medical Academy of Research and Translation (SMART), Shenzhen, 518052, China

**Keywords:** Mitochondrial transplantation, Hydrogel, Peptide, Myocardial infarction, Cardiac repair

## Abstract

Myocardial infarction (MI) is characterized by severe oxidative stress, excessive inflammation, and profound mitochondrial dysfunction. Although mitochondrial transplantation offers therapeutic promise for MI, its clinical translation is severely hampered by the extreme fragility of donor mitochondria with rapid loss of functional viability after isolation. Here, inspired by the intrinsic cellular defense mechanisms against mitochondrial dysfunction, MOTS-c, a mitochondria-derived peptide (MDP), is selected and further conjugated with self-assembling peptide (Q11) to fabricate a hydrogel-based mitochondrial delivery system (MQ^gel^@Mito) for cardiac repair after MI. It has been observed that MQ^gel^ significantly extends the survival of isolated mitochondria and maintains metabolic enzyme activity for at least 8 h. More importantly, MQ^gel^ not only shields donor mitochondria from oxidative stress and calcium overload, but also enhances mitochondrial internalization by macrophages through an adenosine 5′-monophosphate-activated protein kinase (AMPK)-dependent mechanism. Furthermore, MQ^gel^@Mito facilitates metabolic reprogramming of macrophages by suppressing pro-inflammatory glycolysis and enhancing oxidative phosphorylation (OXPHOS), thereby attenuating M1 polarization. Additionally, MQ^gel^@Mito maintains mitochondrial homeostasis, reduces reactive oxygen species (ROS), and rescues apoptosis of macrophages. In a rat MI model, MQ^gel^@Mito reduces M1 macrophage infiltration and cardiomyocyte damage by delivering viable mitochondria, thereby improving cardiac function and limiting pathological remodeling. These findings establish a paradigm for mitochondrial protection and demonstrate macrophage immunometabolism as a viable therapeutic strategy for MI.

## Highlights

• A MOTS-c–peptide hydrogel (MQ^gel^) prolongs isolated mitochondrial viability.
• MQ^gel^ shields isolated mitochondria from oxidative stress and calcium overload.
• MQ enhances macrophage mitochondrial uptake via AMPK-driven macropinocytosis.
• MQ^gel^@Mito suppresses pro-inflammatory glycolysis and restores TCA in macrophages.
• MQ^gel^@Mito attenuates inflammation and improves cardiac remodeling post-MI.

## Graphical abstract

**Figure:**
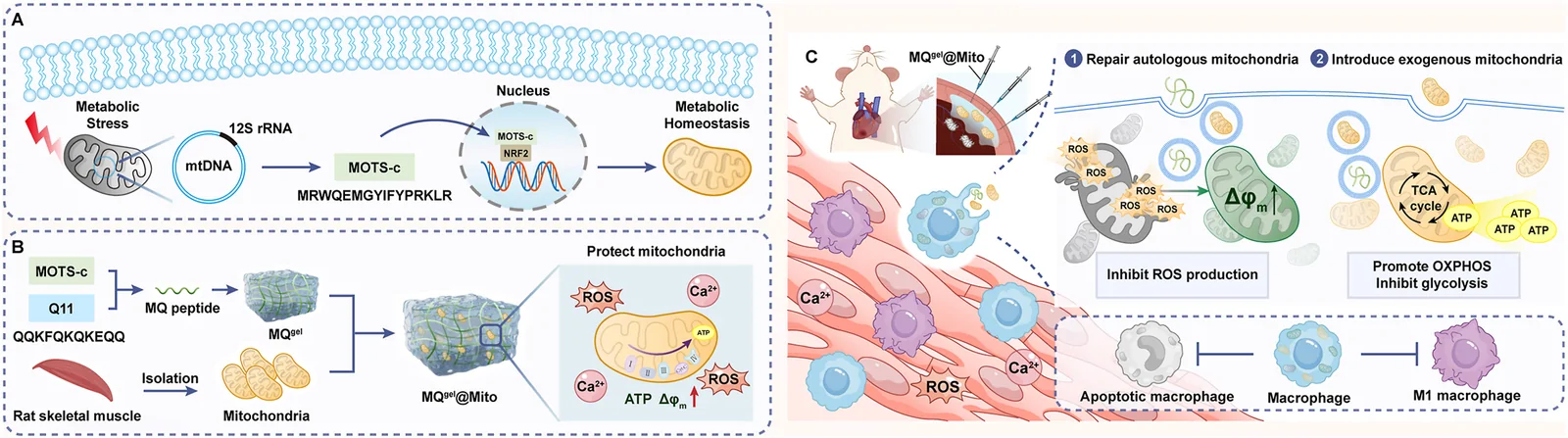

## 1 Introduction

Ischemic heart disease is a leading contributor to global mortality, representing the most prevalent cause of death within the broad spectrum of cardiovascular diseases, which constitute a substantial health burden worldwide [1]. Unlike the repair processes in other tissues, adult cardiomyocytes possess negligible regenerative capacity. Consequently, the healing of damaged myocardial tissue is predominantly mediated by immune cells and fibroblasts, leading to scar formation [2,3]. Among them, macrophages, which originate from both circulating pools and resident populations, are considered the most crucial players in post-injury cardiac repair. They not only exhibit the function of regulating inflammation, but also interact with cardiomyocytes, endothelial cells, and fibroblasts, fundamentally driving post-MI cardiac remodeling [4]. However, factors such as hypoxia, oxidative stress, and metabolic disturbances in the infarcted area severely impair mitochondrial function in macrophages, hindering their transition to the reparative phenotype and thereby delaying and weakening the heart's self-healing capacity [5]. Although recent strategies targeting mitochondrial antioxidation and metabolic modulation have partially restored cellular function [6,7], their therapeutic efficacy is limited in cells with pre-existing mitochondrial dysfunction and damaged mitochondrial DNA (mtDNA) [8]. Therefore, developing interventions that systemically enhance the metabolic adaptability and reparative functions of macrophage mitochondria will represent a key direction in myocardial repair and regenerative therapy.

Mitochondrial transplantation therapy (MTT) is an innovative and promising strategy that delivers the healthy mitochondria either derived from normal cells or reassembled through synthetic biology into the cells and tissues to replace their defective mitochondria and restore their function [9]. Pioneering clinical studies have demonstrated that autologous mitochondrial transplantation improved outcomes in pediatric patients with myocardial ischemia-reperfusion injury, while a recent trial has expanded the therapeutic scope to inflammatory myopathies [10,11]. However, the clinical translation of MTT has been severely constrained by interrelated bottlenecks. First, isolated mitochondria are extraordinarily fragile, losing functional viability within approximately 1 h *in vitro* [12]. Beyond this narrow window, mitochondrial membrane potential dissipates, respiratory chain function deteriorates, and therapeutic efficacy is substantially compromised [13,14]. Second, the infarcted microenvironment, characterized by pathological oxidative stress and calcium overload, rapidly compromises transplanted organelle function and induces permeability transition [15]. Third, the efficiency of naked mitochondrial internalization by recipient cells remains extremely low, leading to substantial wastage and undesired degradation [16]. Although various biomaterials, including hydrogels, microvesicles, and polymeric microcapsules, have been developed to encapsulate and protect donor mitochondria, their protective effects are merely confined to physical shielding and rely on modifications of conventional cell delivery strategies [[17], [18], [19], [20]]. It remains challenging to develop a simple, feasible, and rapidly scalable protective strategy that can effectively preserve mitochondrial structural integrity and improve mitochondrial delivery efficiency.

**Fig. 1:**
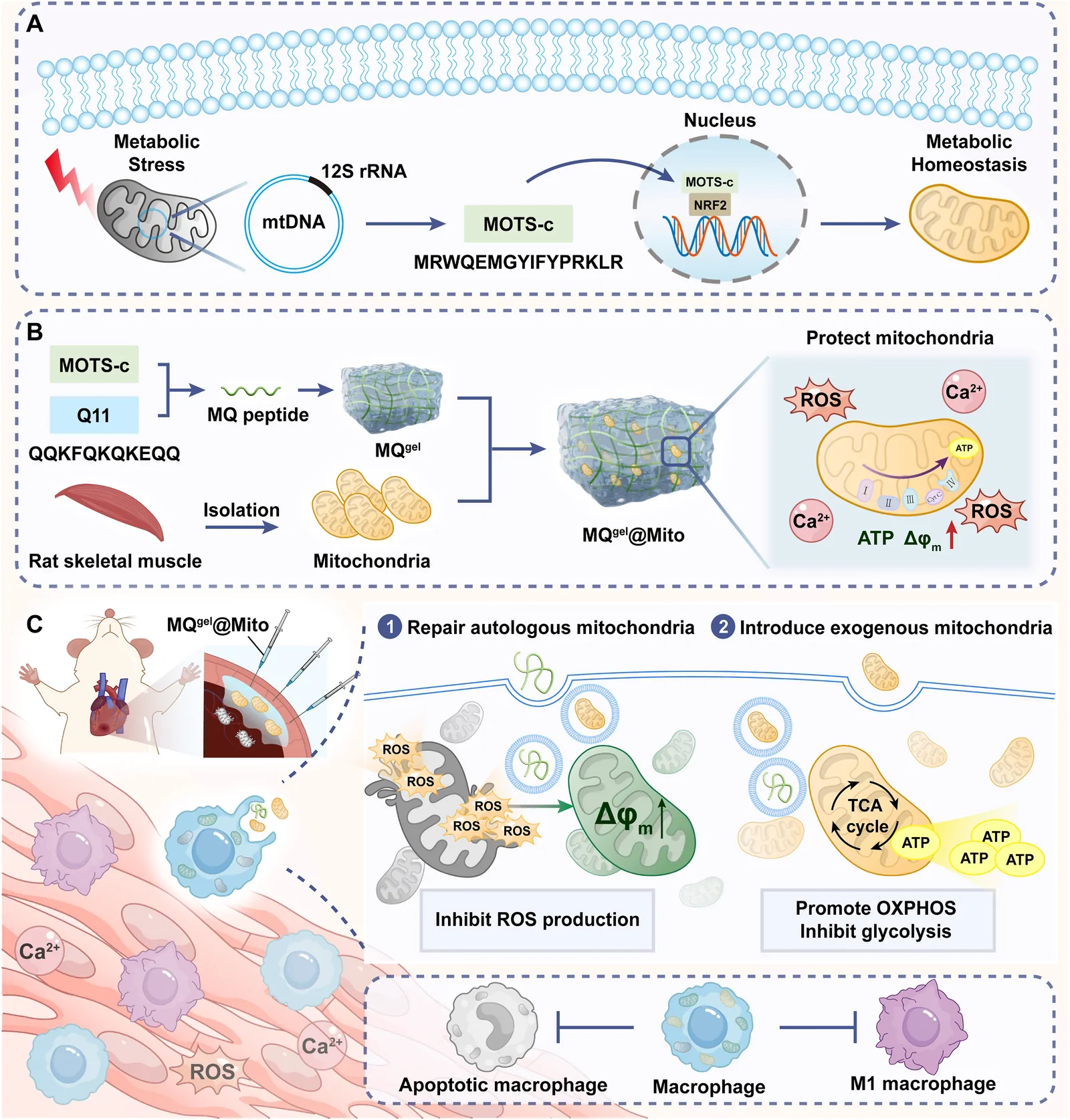
Scheme illustrating the protective effect of MQ^gel^ on donor mitochondria and the therapeutic effect of MQ^gel^@Mitoagainst MI. (A) MOTS-c is a typical MDP and exerts mitochondrial protective functions. (B) The fabrication of MQ^gel^@Mito and the protective effect on isolated donor mitochondria. (C) The therapeutic effect and mechanism of MQ^gel^@Mito on promoting cardiac repair via macrophage metabolic reprogramming.

MDPs, a unique class of peptides encoded by short open reading frames in mtDNA, function as an intrinsic cellular defense mechanism against mitochondrial dysfunction, primarily by enhancing mitochondrial function through the regulation of energy metabolism and antioxidant responses [21]. As a recently identified MDP, Mitochondrial Open Reading Frame of the 12S rRNA Type-C (MOTS-c) promotes systemic metabolic homeostasis mainly by modulating glucose and lipid metabolism and decreasing ROS production, thereby exerting a mitochondrial protective function (Fig. 1A) [22,23]. Inspired by these unique characteristics, we hypothesized that MOTS-c could serve as a potential protective candidate for mitochondrial transplantation; however its application is constrained by its high solubility and low bioavailability [24]. Herein, we further conjugated MOTS-c with Q11 peptide to fabricate a co-assembled hydrogel platform (MQ^gel^) for the delivery and protection of donor mitochondria. The Q11 peptide, a low-molecular-weight peptide with 11 amino acids, was selected for its ability to rapidly self-assemble into elastic hydrogels with tunable mechanical properties and inherent self-healing capability [25]. The isolation of functional mitochondria and their activity protection by MQ^gel^ (referred to as MQ^gel^@Mito) were systematically characterized (Fig. 1B). Furthermore, this study explored the internalization of donor mitochondria and the regulatory effects of MQ^gel^@Mito on macrophage phenotypic transformation. Moreover, metabolic profiling analysis was conducted to reveal the mechanism of MQ^gel^@Mito-induced inflammatory phenotype transition of macrophages. Besides, we evaluated the influence of MQ^gel^@Mito on the mitochondrial homeostasis and cellular apoptosis of macrophages under oxidative stress. Additionally, the therapeutic effects of MQ^gel^@Mito on cardiac function in rats with MI were evaluated (Fig. 1C).

## 2 Results

### 2.1 Isolation of functional mitochondria and fabrication of MQ^gel^@Mito

**Fig. 2:**
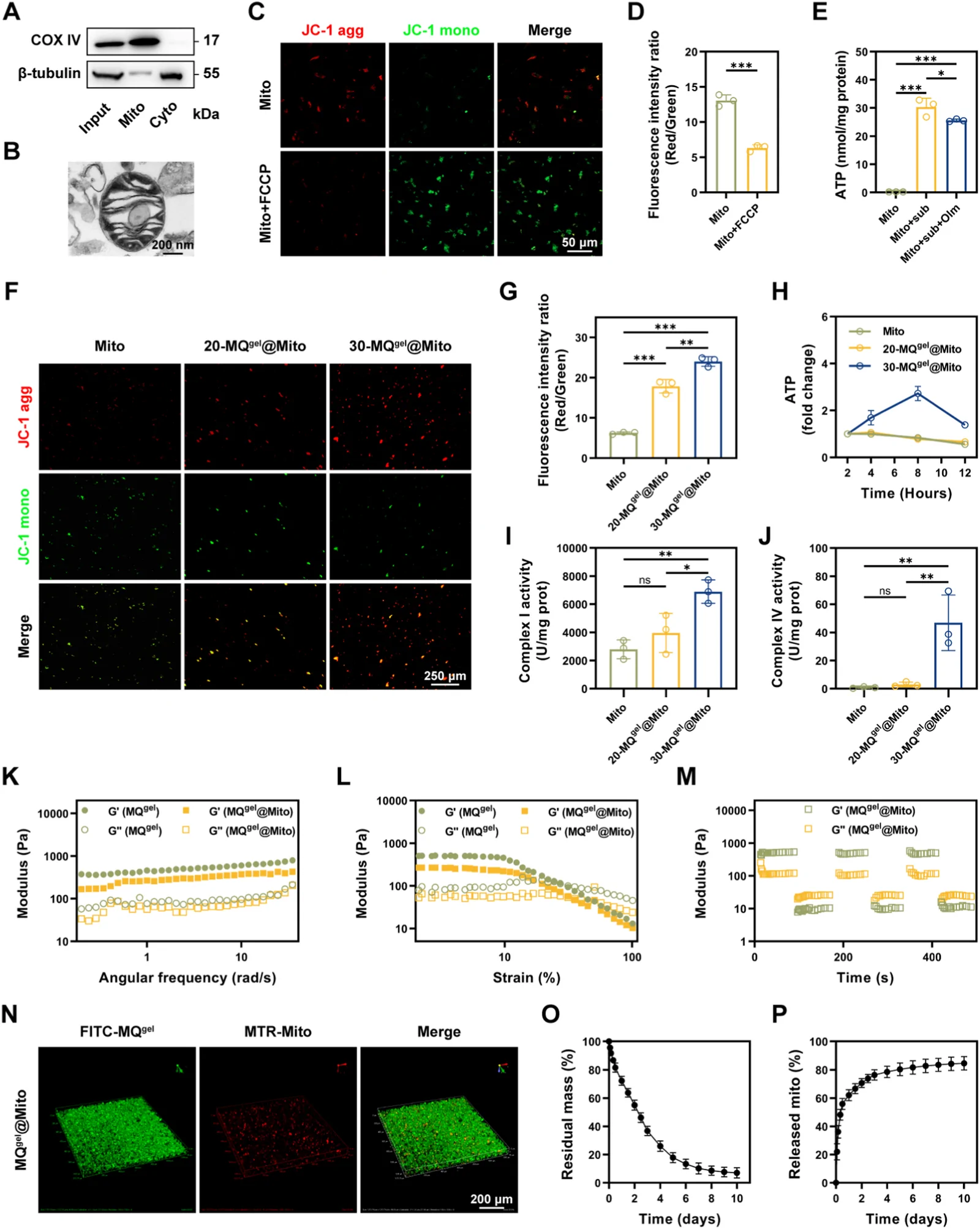
Isolation of functional mitochondria and fabrication of MQ^gel^@Mito. (A) Expression levels of COX Ⅳ and β-tubulin were evaluated in input, isolated mitochondria suspension (Mito), and cytoplasm (Cyto) to assess the purity of the isolated mitochondria. (B) Representative TEM image of isolated mitochondria. (C) Representative fluorescence images of JC-1 staining in isolated mitochondria. Agg, JC-1 aggregates (red); Mono, JC-1 monomers (green). (D) Quantification of red/green ratio of JC-1 staining in isolated mitochondria (n = 3). (E) ATP production of isolated mitochondria (n = 3). Sub, respiratory substrates; Oly, oligomycin. (F) Representative JC-1 staining fluorescence images of mitochondria at 8 h post-isolation. (G) JC-1 fluorescence intensity ratio (red/green) of mitochondria at 8 h post-isolation (n = 3). (H) Relative fold change in ATP levels of isolated mitochondria protected by MQ^gel^ at different concentrations (n = 3). (I-J) Activity of mitochondrial respiratory chain Complex Ⅰ (I) and Complex Ⅳ (J) of isolated mitochondria protected by MQ^gel^ at different concentrations (n = 3). (K) Rheological properties of 30 mg mL^−1^ MQ^gel^ and MQ^gel^@Mito under frequency sweeps ranging from 0.1 to 34 rad s^−1^. G′ represents storage modulus; G′′ represents loss modulus. (L) Rheological properties of 30 mg mL^-1^ MQ^gel^ and mg mL^-1^ MQ^gel^@Mito under strain sweeps. (M) Self-healing properties of 30 mg mL^-1^ MQ^gel^@Mito under alternating oscillatory strains: high strain (100%) and low strain (1%). (N) Representative 3D z-stack confocal fluorescence images of MQ^gel^@Mito. (O) The degradation profile of MQ^gel^@Mito *in vitro* (n = 3). (P) Mitochondrial release kinetics from MQ^gel^@Mito *in vitro* (n = 3). Data are presented as mean ± standard deviation (SD); ns, not significant; *p < 0.05, **p < 0.01, ***p < 0.001.

We isolated donor mitochondria from the skeletal muscles of Sprague Dawley rats. To ensure the success of mitochondrial transplantation, the quality of isolated mitochondria was strictly detected using different methods. Western blot analysis confirmed the purity of the isolated naked mitochondria (Mito), with robust expression of the mitochondrial marker cytochrome *c* oxidase subunit Ⅳ (COX Ⅳ) and the absence of the cytoplasmic marker β-tubulin (Fig. 2A). Transmission electron microscopy (TEM) revealed characteristic double-membrane ultrastructure with well-organized cristae, indicating preserved morphological integrity (Fig. 2B). Functional assessment demonstrated that, immediately after isolation, the naked mitochondria exhibited strong respiratory competence. JC-1 staining confirmed high mitochondrial membrane potential (ΔΨm), with the naked mitochondria displaying predominantly red fluorescence (JC-1 aggregates) indicative of polarized organelles, which could be converted by the mitochondrial uncoupler carbonyl cyanide-4-(trifluoromethoxy) phenylhydrazone (FCCP) to green fluorescence (JC-1 monomers) emitting principally (Fig. 2C and D). The synthesis of ATP, relying on the relatively high ΔΨm, directly reflects the functional viability of mitochondria, as evidenced by substantial ATP production in the presence of respiratory substrates, which was abolished upon addition of the ATP synthase inhibitor oligomycin (Fig. 2E). These quality control measures collectively established that our isolation protocol yielded structurally intact and functionally active mitochondria suitable for transplantation studies.

Subsequently, MQ^gel^ was fabricated by covalently conjugating the mitochondria-derived peptide MOTS-c (MRWQEMGYIFYPRKLR) with the self-assembling peptide Q11 (QQKFQFQFEQQ) at a 1:1 stoichiometric ratio, after which an appropriate amount of NaCl was added to the peptide solution to induce the formation of the hydrogel. The circular dichroism (CD) spectra revealed that MOTS-c exhibited a random coil conformation, evidenced by a negative band near 200 nm. In contrast, Q11 displayed a characteristic β-sheet signature with a negative band at around 216 nm. The MQ peptide exhibited a mixed CD profile containing both α-helical and antiparallel β-sheet contributions (Fig. S1A). Deconvolution of the spectra indicated that the antiparallel β-sheet content accounted for 52.3% of the total secondary structure, confirming that the antiparallel β-sheet conformation, the structural prerequisite for self-assembly, was preserved after MOTS-c conjugation (Fig. S1B). The TEM images further demonstrated that both Q11 and MQ formed well-defined nanofibrous networks, whereas MOTS-c did not assemble into ordered structures under the same conditions (Fig. S2).

We fabricated MQ^gel^ at concentrations of 20, 30, and 40 mg mL^−1^ as described in a previously published protocol (Fig. S3) [26]. The mechanical properties of MQ^gel^ were characterized via rheological testing, and the storage modulus (G′) values exceeded the loss modulus (G″) across all concentrations in the angular frequency sweep testing, confirming successful hydrogel formation (Fig. S4). Given that the storage modulus of 40 mg mL^−1^ MQ^gel^ exceeded 1000 Pa, which potentially limited the intramyocardial injectability, we continued to investigate the protective effects of 20 and 30 mg mL^−1^ MQ^gel^ via functional assessments of mitochondria. At 8 h post-isolation, JC-1 staining revealed that mitochondria encapsulated in 30 mg mL^−1^ MQ^gel^ exhibited the strongest red fluorescence and had a significantly higher red/green fluorescence ratio than the other groups, while naked mitochondria showed the lowest ratio (Fig. 2F and G). Consistently, ATP synthesis assays demonstrated that 30 mg mL^−1^ MQ^gel^ sustained mitochondrial ATP production for up to 8 h — a marked extension over unprotected mitochondria — whereas 20 mg mL^−1^ MQ^gel^ had little effect. Beyond 8 h, all groups exhibited irreversible loss of synthetic capacity, defining the practical storage window (Fig. 2H). Assessment of respiratory chain complex activities further confirmed that 30 mg mL^−1^ MQ^gel^ preserved both Complex Ⅰ and Complex Ⅳ function after 8 h (Fig. 2I and J). According to these results, 30 mg mL^−1^ MQ^gel^ was selected for mitochondrial protection. To distinguish the contributions of Q11^gel^, MOTS-c, or MQ^gel^ to mitochondrial protection, we compared their effects on ATP synthesis of isolated mitochondria at the 8-h time point. All three treatments improved ATP production relative to naked mitochondria, with MQ^gel^ displaying the strongest protective effect, as reflected by the highest ATP synthesis activity (Fig. S5).

Rheological characterization of the composite system (MQ^gel^@Mito) demonstrated that G′ consistently exceeded G″ across the angular frequency range tested, confirming elastic gel-like behavior (Fig. 2K, Fig. S6). Strain sweep testing revealed shear-thinning properties essential for injection through narrow-gauge needles (Fig. 2L). In time-sweep rheological tests, the values of G′ exceeded those of modulus (G″) at a low strain of 1%, but fell below G″ at a high strain of 100%. This strain-dependent viscoelastic behavior demonstrated the self-healing capability of both MQ^gel^@Mito and MQ^gel^, a critical property for maintaining structural integrity after injection into cardiac tissue (Fig. 2M; Fig. S7). To directly visualize the distribution of mitochondria within the hydrogel, MQ^gel^ was labeled with fluorescein isothiocyanate (FITC) for green fluorescence, and mitochondria were labeled with MitoTracker Red (MTR) for red fluorescence. Three-dimensional confocal z-stack scanning of the resulting MQ^gel^@Mito provided direct visualization of the isolated mitochondria encapsulated within the hydrogel matrix rather than merely on its surface (Fig. 2N). Scanning electron microscopy (SEM) revealed that 30 mg mL^−1^ MQ^gel^ exhibited a porous three-dimensional architecture resembling an extracellular matrix scaffold (Fig. S8). A gravimetric degradation assay showed that MQ^gel^@Mito underwent gradual degradation *in vitro* in approximately 10 days at 37 °C (Fig. 2O). The mitochondrial release profile closely paralleled the hydrogel degradation kinetics, with 78.6% ± 4.1% of encapsulated mitochondria released by day 4, after which the release rate slowed substantially and ceased by day 10 (Fig. 2P). This concordance between degradation and release confirmed that mitochondrial release was governed by hydrogel matrix erosion and that MQ^gel^@Mito could achieve sustained mitochondrial delivery. Overall, these results demonstrated that we successfully isolated functional mitochondria with intact ultrastructure and biological function, and fabricated a hydrogel system for the protection of isolated mitochondria (MQ^gel^@Mito).

### 2.2 MQ^gel^ shielded isolated mitochondria from oxidative stress and calcium overload

**Fig. 3:**
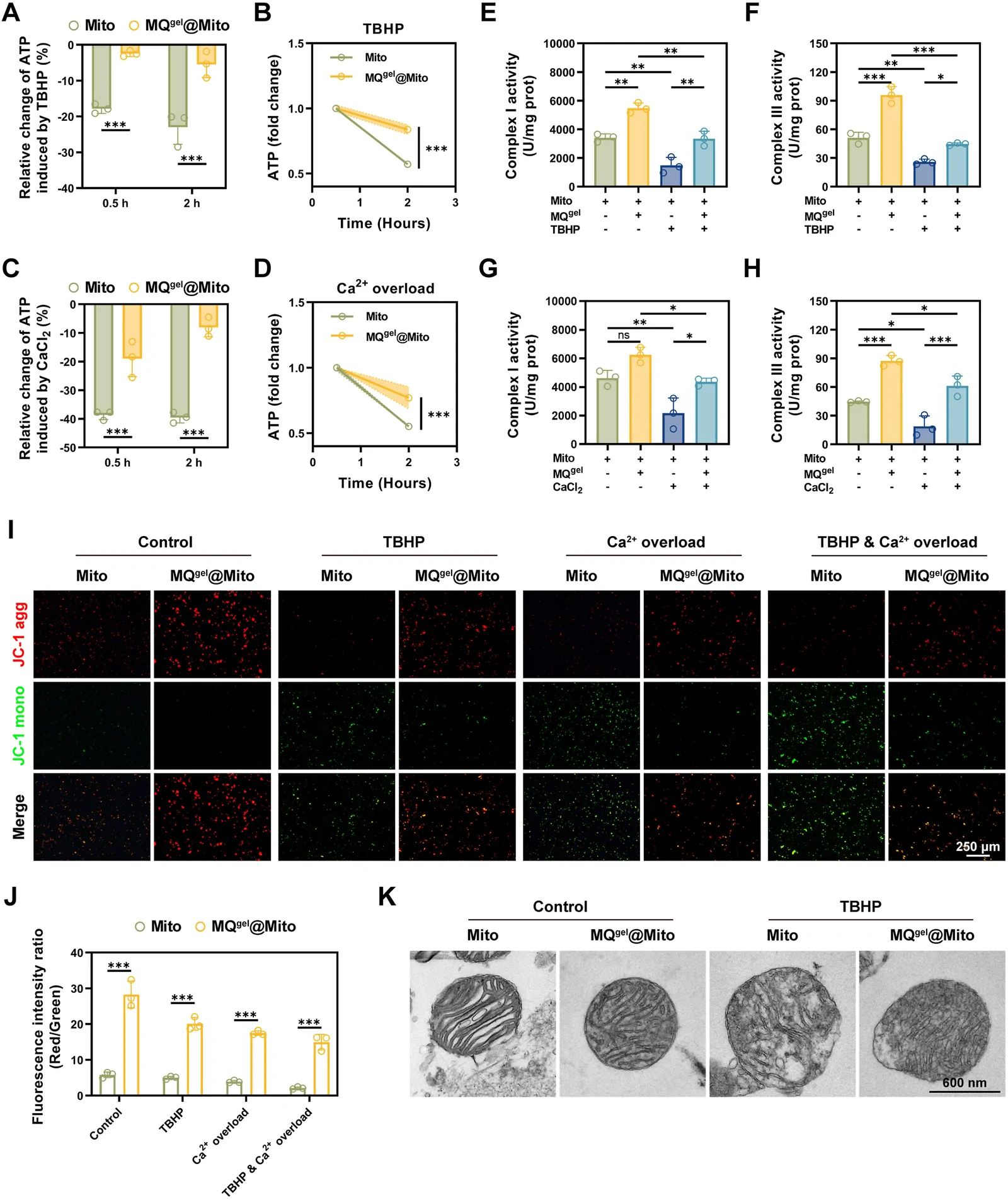
MQ^gel^ shields isolated mitochondria from oxidative stress and calcium overload. (A) ATP relative reduction induced by TBHP at 0.5 and 2 h (n = 3). (B) Fold change of ATP production under TBHP exposure from 0.5 to 2 h (n = 3). (C) ATP relative reduction induced by calcium overload at 0.5 and 2 h (n = 3). (D) Fold change of ATP production under calcium overload from 0.5 to 2 h (n = 3). (E-F) Activity of mitochondrial respiratory chain Complex I and Complex III in isolated mitochondria with or without TBHP exposure (n = 3). (G-H) Activity of mitochondrial respiratory chain Complex I and Complex III in isolated mitochondria with or without calcium overload (n = 3). (I) Representative JC-1 staining fluorescence images of mitochondria under different stimuli. (J) Fluorescence intensity ratio (red/green) of mitochondria stained with JC-1 (n = 3). (K) Representative TEM images of isolated mitochondria under TBHP exposure. Data are presented as mean ± SD; ns, not significant; *p < 0.05, **p < 0.01, ***p < 0.001.

The post-infarction myocardial microenvironment presents formidable challenges to transplanted mitochondria, characterized by pathological accumulation of ROS and calcium overload, both of which severely compromise organelle function. We therefore evaluated the protective capacity of MQ^gel^ under conditions simulating these stressors, using tert-butyl hydroperoxide (TBHP) to model oxidative stress and CaCl_2_ to simulate calcium overload, respectively. First, we assessed the functional consequences, which were striking: compared with untreated mitochondria, naked mitochondria exposed to TBHP showed a 17.85% ± 1.30% reduction in ATP production at 30 min, and the reduction increased to 22.99% ± 4.76% at 2 h. In contrast, MQ^gel^@Mito exhibited strong resistance to TBHP-induced damage, with ATP production decreasing by merely 2.46% ± 0.81% and 5.46% ± 3.78% at the two corresponding time points (Fig. 3A). Longitudinal analysis with data normalized to the 30-min baseline confirmed that MQ^gel^@Mito exerted sustained protective effects over time. After 2 h of TBHP exposure, ATP synthesis in MQ^gel^@Mito retained 0.84 of its 30-min level, whereas unprotected mitochondria retained only 0.57 (Fig. 3B). Calcium overload also induced distinct differences between groups. Naked mitochondria had ATP synthesis reduced by 38.63% ± 1.66% (30 min) and 39.55% ± 1.87% (2 h), while the reductions for MQ^gel^@Mito were merely 19.00% ± 6.30% and 8.06% ± 3.31% (Fig. 3C). Two hours post calcium overload, ATP levels in MQ^gel^-protected and unprotected mitochondria fell to 0.77- and 0.55-fold of their 30-min values (Fig. 3D).

To elucidate the mechanistic basis of this protection, we assessed the activities of mitochondrial respiratory chain complexes. Exposure to either stressor markedly inhibited respiratory chain function of naked mitochondria, yet MQ^gel^ substantially preserved the activities of Complex Ⅰ and Complex Ⅲ under both conditions (Fig. 3E–H). Besides, we detected the mitochondrial membrane potential using JC-1 staining. TBHP exposure caused substantial depolarization, evidenced by diminished red fluorescence and enhanced green fluorescence, an effect largely reversed by MQ^gel^, indicating the preserved membrane potential. Similar protective effects were observed under calcium overload alone, as well as under combined TBHP and calcium overload, demonstrating that MQ^gel^ consistently protects mitochondria across these adverse conditions (Fig. 3I and J). Quantification of mitochondrial superoxide using MitoSOX Red demonstrated significantly lower fluorescence intensity in MQ^gel^@Mito compared to naked mitochondria following TBHP exposure (Fig. S9). From a structural perspective, TEM revealed that TBHP induced morphological abnormalities such as mitochondrial vacuolization and cristae disruption, which were partially rescued by MQ^gel^ encapsulation (Fig. 3K). Overall, these findings indicated that MQ^gel^ constructed a favorable microenvironment to maintain mitochondrial homeostasis under adverse conditions encountered during mitochondrial transplantation, such as elevated oxidative stress and calcium overload.

### 2.3 MQ-mediated mitochondrial internalization modulated macrophage polarization and improved anti-inflammatory responses

**Fig. 4:**
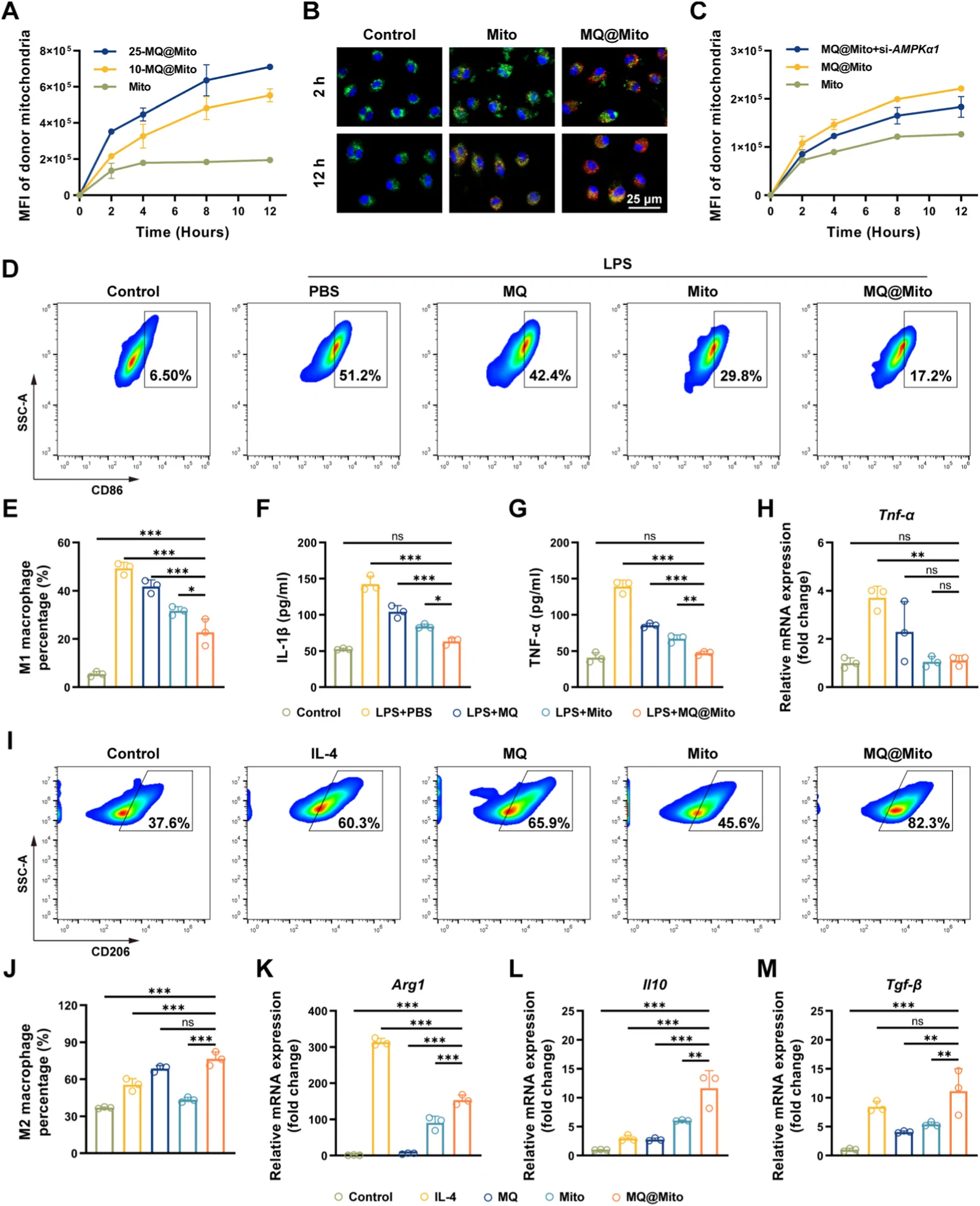
MQ-mediated mitochondrial internalization modulates macrophage polarization and improves anti-Inflammatory responses. (A) Time-course analysis of donor mitochondrial uptake in BMDMs. Cells were treated with Mito, 10-MQ@Mito (MQ, 10 μM), or 25-MQ@Mito (MQ, 25 μM). Uptake was quantified by flow cytometry based on the MFI of MTR-labeled mitochondria (n = 3). MFI, mean fluorescence intensity; MTR, MitoTracker Red. (B) Colocalization of MTG-labeled recipient mitochondria (green) and MTR-labeled donor mitochondria (red) in BMDMs after 2 h and 12 h of mitochondria transplantation. MTG, MitoTracker Green. Cell nuclei were counterstained with Hoechst (blue). (C) Time-course analysis of donor mitochondrial uptake in BMDMs. Cells were treated with Mito, MQ@Mito, or MQ@Mito+si-*AMPKα1*. Uptake was quantified by flow cytometry based on the MFI of MTR-labeled mitochondria (n = 3). (D) Representative flow cytometry scatter plots showing the proportion of F4/80^+^CD86^+^ M1 macrophages under different treatments. (E) Quantification of the proportion of F4/80^+^CD86^+^ M1 macrophages (n = 3). (F-G) ELISA analysis of pro-inflammatory cytokines IL-1β and TNF-α in the culture supernatant (n = 3). (H) qPCR analysis of *Tnf-α* mRNA expression (n = 3). (I) Representative flow cytometry plots showing F4/80^+^CD206^+^ M2 macrophages under different treatments. (J) Quantification of the percentage of F4/80^+^CD206^+^ M2 macrophages (n = 3). (K-M) qPCR analysis of M2-associated marker genes *Arg1*, *Il10*, and *Tgf-β* (n = 3). Data are presented as mean ± SD; ns, not significant; *p < 0.05, **p < 0.01, ***p < 0.001.

Macrophages have emerged as central orchestrators of the post-MI response, governing the transition from acute inflammation to tissue repair. Therefore, macrophages were applied as recipient cells and the donor mitochondrial internalization was first investigated. *In vitro*, MQ peptide solution was used to mimic the degradation-released functional products of MQ^gel^. CCK-8 and live/dead staining demonstrated that MQ exhibited excellent biocompatibility with bone marrow-derived macrophages (BMDMs) (Fig. S10). Additionally, fluorescence imaging further confirmed that MQ could be internalized by BMDMs (Fig. S11). Subsequently, we incubated BMDMs with MTR-labeled donor mitochondria in the presence of MQ with different concentrations (0, 10, and 25 μM) for several periods. Flow cytometry analysis revealed that MQ significantly enhanced donor mitochondrial internalization compared with naked mitochondria, as evidenced by increased mean fluorescence intensity (MFI) of MTR (Fig. S12). Quantitative analysis of MFI revealed a time-dependent rise in MFI in BMDMs. Notably, high-concentration MQ markedly promoted the internalization of donor mitochondria (Fig. 4A). Fluorescence images confirmed efficient cytoplasmic colocalization of donor and recipient mitochondria following co-incubation with high-concentration MQ (Fig. 4B).

We next investigated the underlying molecular mechanism that MQ enhanced mitochondrial internalization. Evidence has been found that mitochondria can be internalized via macropinocytosis, a low-efficiency, non-receptor-mediated process [16]. Macrophages, as immune cells, exhibit high levels of macropinocytic activity, which is closely connected with energy metabolism [27]. AMPK acts as a cellular energy sensor and drives the cytoskeletal remodeling necessary for macropinosome formation by activating the actin-severing protein cofilin, thereby enhancing macropinocytosis [[27], [28], [29]]. Meanwhile, it is reported that MOTS-c, the degradation product of MQ, is an MDP that activates AMPK to regulate metabolic homeostasis [21]. Here, we hypothesized the mechanism by which MQ facilitated the internalization of donor mitochondria was related to AMPK activation. AMPK is a heterotrimeric complex (α, β, γ subunits), with the α subunit (α1/α2 isoforms) as the catalytic core [30]. qPCR analysis showed that *AMPKα1* was much more highly expressed than *AMPKα2* in BMDMs (Fig. S13A), so we constructed *AMPKα1*-specific siRNA (si-*AMPKα1*). Both qPCR and western blot verified significant si-*AMPKα1* knockdown at mRNA and protein levels (Fig. S13B–D). We then assessed the effect of *AMPKα1* knockdown on mitochondrial uptake in BMDMs at different time points by quantifying the MFI of MTR-labeled mitochondria. Mitochondrial uptake was persistently higher in the MQ@Mito group relative to the Mito group. This promoting effect was largely abrogated following *AMPKα1* silencing (Fig. 4C, Fig. S14). Western blot analysis further validated the mechanism. Compared with the Mito group, the MQ@Mito group showed elevated p-AMPKα/AMPKα ratio. Such upregulation was obviously reversed upon *AMPKα1* knockdown (Fig. S15) and the AMPK inhibitor Compound C (CC) treatment (Fig. S16). To directly confirm the involvement of macropinocytosis, we performed a FITC-dextran uptake assay. MQ@Mito elevated the MFI of FITC-dextran in BMDMs, reflecting enhanced macropinocytic activity, which was abolished by si-*AMPKα1* (Fig. S17). In addition, MQ also significantly promoted mitochondrial uptake in lipopolysaccharide (LPS)-pretreated M1 macrophages and this effect was completely abrogated by si-*AMPKα1* (Fig. S18). Collectively, these results demonstrated that MQ promoted mitochondrial uptake by activating AMPK-mediated macropinocytosis.

Given the emerging role of mitochondria in regulating macrophage phenotype, we next examined whether MQ@Mito could modulate macrophage polarization [31,32]. LPS-stimulated BMDMs induced robust M1 polarization, characterized by elevated CD86 expression. MQ@Mito pretreatment visually attenuated this pro-inflammatory phenotype, as evidenced by diminished CD86 immunofluorescence (Fig. S19) and a significant reduction in the F4/80^+^CD86^+^ population (Fig. 4D and E). To further evaluate the therapeutic potential of MQ@Mito beyond preventive intervention, we examined its effect on M1 polarization under two distinct administration regimens: co-treatment (MQ@Mito added simultaneously with LPS), and post-treatment (MQ@Mito added after LPS-induced M1 polarization). Flow cytometry analysis revealed that MQ@Mito significantly reduced the proportion of F4/80^+^CD86^+^ M1 macrophages under these two conditions (Fig. S20), suggesting that MQ@Mito can reverse, rather than merely prevent, the established pro-inflammatory macrophage phenotype. Furthermore, ELISA measurements of supernatant revealed suppressed secretion of pro-inflammatory cytokines including interleukin-1β (IL-1β) and tumor necrosis factor (TNF-α) (Fig. 4F and G). In line with these findings, qPCR analysis of BMDMs demonstrated reduced *Tnf-α* mRNA expression (Fig. 4H). In parallel, MQ@Mito significantly increased the proportion of F4/80^+^CD206^+^ M2 macrophages (Fig. 4I and J), and upregulated expression of *Arg1*, *il10* and *Tgf-β* mRNA (Fig. 4K–M), demonstrating that MQ@Mito treatment could promote M2 polarization. Taken together, these results demonstrated that MQ@Mito effectively suppressed LPS-induced M1 polarization and enhanced M2 polarization.

### 2.4 MQ@Mito modulated macrophage polarization via metabolic reprogramming

**Fig. 5:**
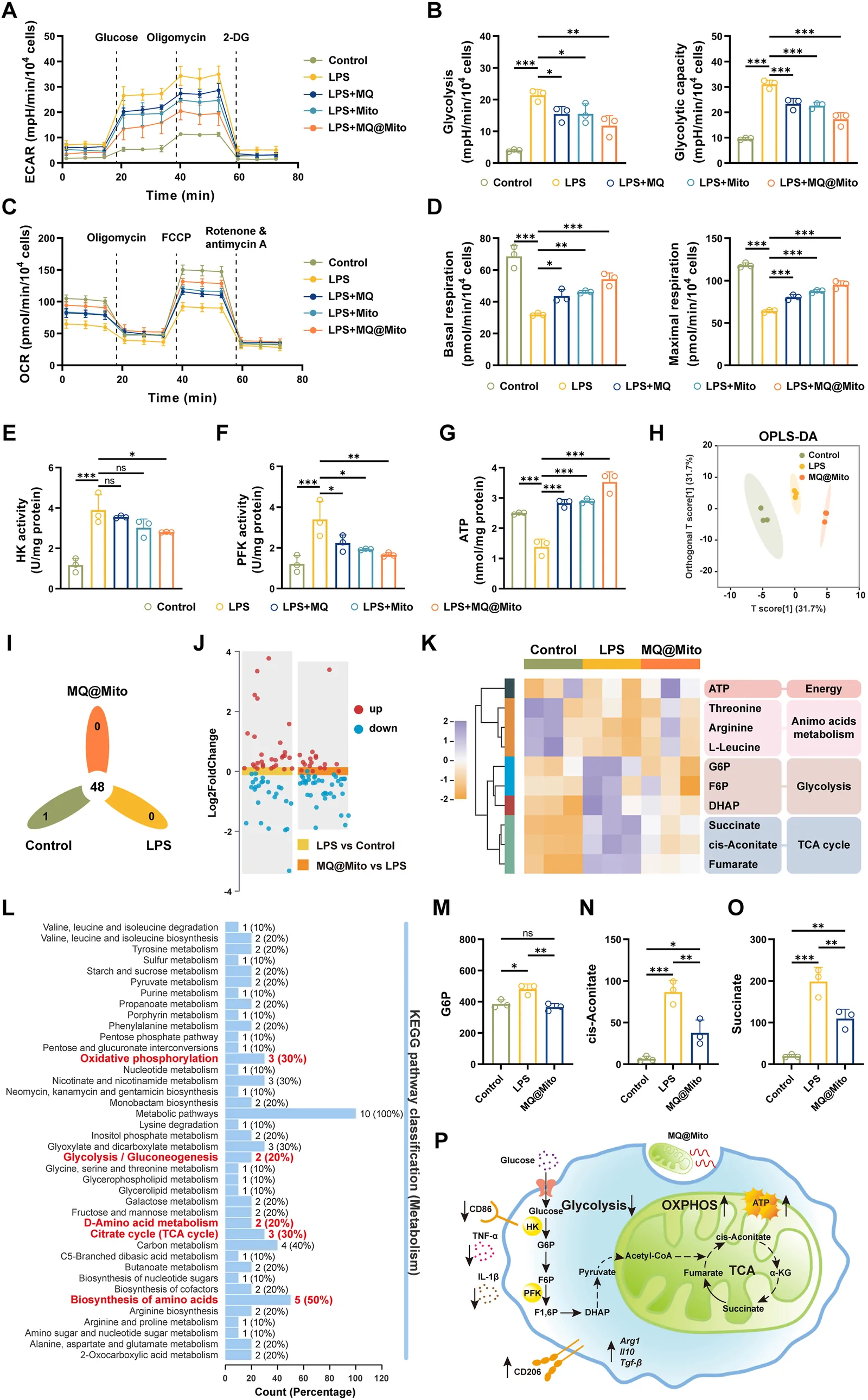
MQ@Mito modulates macrophage polarization by metabolic reprogramming. (A) ECAR determined by the glycolysis stress test using Seahorse (n = 3). (B) Quantitative analysis of glycolysis and glycolytic capacity derived from glycolysis stress test (n = 3). (C) OCR determined by the mitochondrial stress test using Seahorse (n = 3). (D) Quantitative analysis of basal and maximal mitochondrial respiration (n = 3). (E-F) Activity of critical glycolytic enzymes HK (E) and PFK (F) of BMDMs with different treatments (n = 3). HK, hexokinase; PFK, phosphofructokinase. (G) ATP production of BMDMs with different treatments (n = 3). (H) OPLS-DA of metabolic profiles among the Control, LPS, and MQ^gel^@Mito groups (n = 3). (I) Venn diagram illustrating the unique and overlapping metabolites among the Control, LPS, and MQ^gel^@Mito groups. (J) Volcano plot showing metabolites with abundance changes. The red dots refer to the upregulated metabolites, and the blue dots refer to the downregulated metabolites. (K) Clustering heatmap of representative DEMs. (L) Metabolism-related KEGG pathway enrichment analysis of representative DEMs. (M) Abundance of glycolysis-related metabolite (G6P) (n = 3). G6P, D-Glucose-6-phosphate. (N-O) Abundance of TCA-related metabolites cis-aconitate (N) and succinate (O) (n = 3). (P) Scheme illustrating that MQ@Mito regulated the polarization of BMDMs by modulating the metabolic reprogramming. Data are presented as mean ± SD; ns, not significant; *p < 0.05, **p < 0.01, ***p < 0.001.

Accordingly, the immunometabolism of macrophages has garnered significant interest, with studies highlighting subtype-specific pathways that dictate both their phenotype and function, far beyond mere energy provision. Pro-inflammatory macrophages (M1) and anti-inflammatory macrophages (M2) exhibit divergent metabolic programs: the former rely on enhanced glycolysis with impaired OXPHOS, while the latter are characterized by an intact tricarboxylic acid (TCA) cycle and enhanced mitochondrial OXPHOS [33]. In addition, mitochondrial dysfunction in macrophages exacerbates the post-MI inflammatory response and limits regenerative capacity through mechanisms involving oxidative stress [5]. These reports collectively suggest that macrophage immunometabolism could be a potential alternative therapeutic target for MI treatment. Metabolic reprogramming has emerged as a defining feature of macrophage activation states [33]. To elucidate the impacts induced by MQ@Mito on the cellular energy metabolism, we next investigated the effect of MQ@Mito on the metabolic reprogramming of LPS-stimulated macrophages using Seahorse extracellular flux analysis, which allows simultaneous assessment of glycolysis and mitochondrial respiration. In the glycolysis stress test, LPS stimulation significantly increased the extracellular acidification rate (ECAR), indicating a marked shift toward enhanced glycolysis (Fig. 5A). Notably, MQ@Mito treatment exerted the most pronounced inhibitory effect, significantly reducing both glycolysis and glycolytic capacity (Fig. 5B). Conversely, the mitochondrial stress test demonstrated that LPS exposure severely impaired mitochondrial respiration, as reflected by reduced basal and maximal oxygen consumption rates (OCR), while MQ@Mito treatment resulted in the most robust recovery of both basal and maximal respiration (Fig. 5C and D). Consistent with the Seahorse data, the activities of the key glycolytic enzymes hexokinase (HK) and phosphofructokinase (PFK) were significantly upregulated in LPS-stimulated macrophages. This LPS-induced increase in glycolytic enzyme activity was attenuated by treatment with MQ or mitochondria alone, and most effectively suppressed by MQ@Mito (Fig. 5E and F). Furthermore, LPS stimulation led to a significant reduction in intracellular ATP levels, which was most potently restored by MQ@Mito treatment (Fig. 5G).

In addition, to elucidate the metabolic basis of MQ@Mito-mediated anti-inflammatory effects, we further performed targeted energy metabolomics analysis using liquid chromatography-tandem mass spectrometry (LC-MS/MS). Orthogonal projections to latent structures-discriminant analysis (OPLS-DA) revealed clear separation among the Control, LPS, and LPS + MQ@Mito (abbreviated as MQ@Mito) groups, confirming substantial metabolic remodeling (Fig. 5H). The Venn diagram illustrated the unique and overlapping metabolites across the three groups, showing that a total of 48 energy metabolism-related metabolites were detected (Fig. 5I). Volcano plot analysis identified significantly up- and down-regulated metabolites (Fig. 5J). Hierarchical clustering heatmap of representative differentially expressed metabolites (DEMs) demonstrated that MQ@Mito treatment substantially reversed LPS-induced metabolic alterations (Fig. 5K). Metabolism-related Kyoto Encyclopedia of Genes and Genomes (KEGG) pathway classification analysis was performed on these representative DEMs (Fig. 5L). The heatmap and KEGG pathway classification both confirmed the involvement of glycolysis/gluconeogenesis, TCA cycle, oxidative phosphorylation pathways, amino acid biosynthesis, and D-amino acid metabolism. Specific metabolic intermediates were further examined to reveal the mechanistic insights of MQ@Mito. LPS stimulation elevated the abundance of D-glucose-6-phosphate (G6P), a key glycolytic intermediate, which was normalized by MQ@Mito treatment (Fig. 5M). Analysis of TCA cycle intermediates revealed that LPS disrupted the cycle, causing accumulation of cis-aconitate and succinate, while MQ@Mito treatment alleviated this metabolic disruption (Fig. 5N and O). The mechanistic basis likely involved dual contributions: the MQ peptide, as an MDP, enhanced mitochondrial function and promoted metabolic normalization, while exogenous healthy mitochondria directly augmented cellular OXPHOS capacity. In summary, MQ@Mito exerted anti-inflammatory effects by reprogramming macrophage metabolism, specifically reversing the LPS-induced glycolytic shift while restoring TCA cycle integrity and OXPHOS levels (Fig. 5P).

### 2.5 MQ@Mito preserved mitochondrial homeostasis and attenuated oxidative injury

**Fig. 6:**
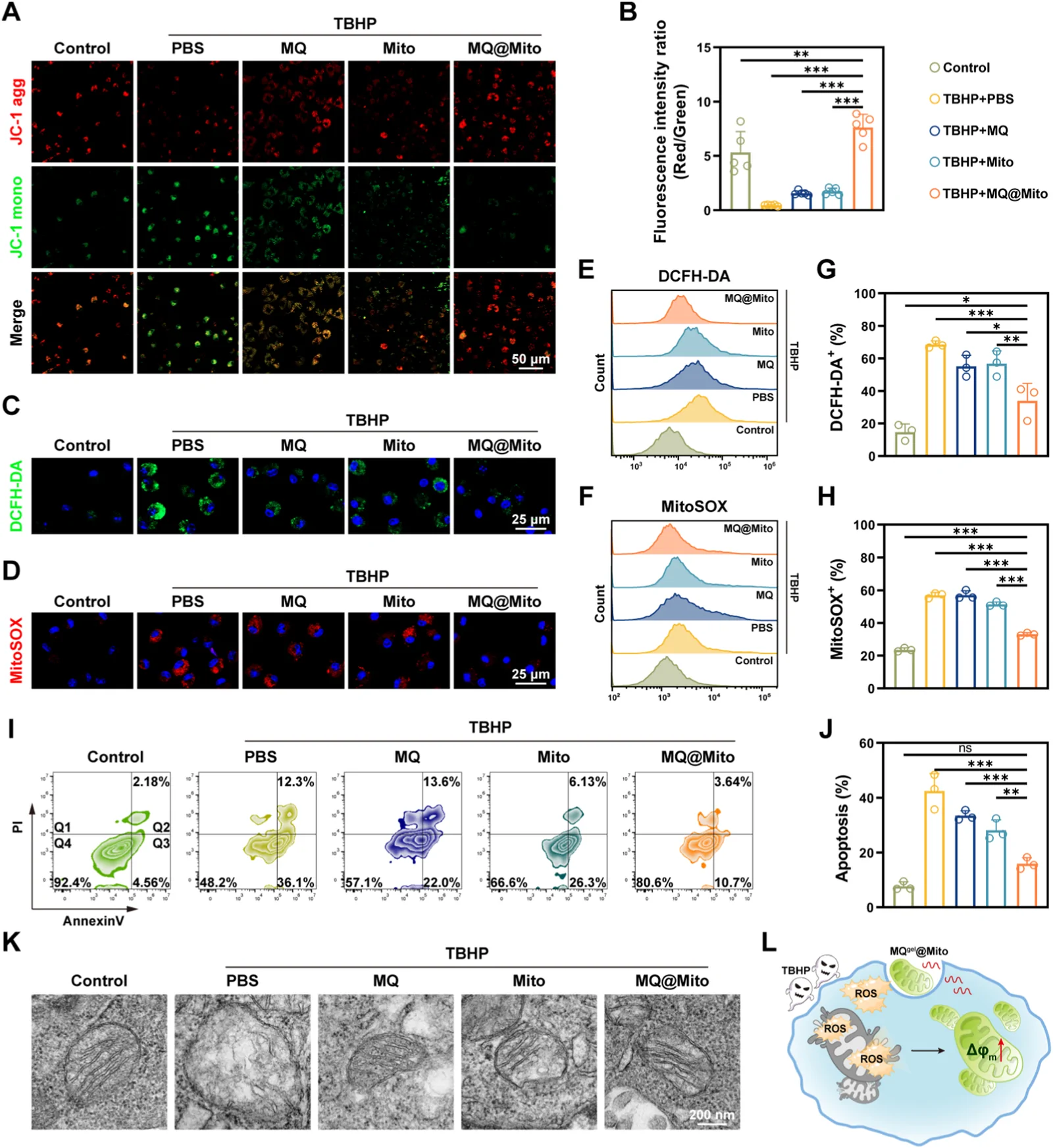
MQ@Mito preserves mitochondrial homeostasis and attenuates oxidative injury. (A) Representative JC-1 fluorescence images of BMDMs after different treatments. (B) Quantification of the red/green JC-1 fluorescence intensity ratio of BMDMs after different treatments (n = 5). (C) Representative fluorescence images of cROS levels in BMDMs, stained with DCFH-DA (green) after different treatments. Cell nuclei were counterstained with Hoechst (blue). (D) Representative fluorescence images of mtROS levels in BMDMs, stained with MitoSOX (red) after different treatments. Cell nuclei were counterstained with Hoechst (blue). (E-F) Representative flow cytometry histograms of DCFH-DA (E) and MitoSOX (F) levels in BMDMs after different treatments. (G-H) Quantitative analysis of MFI of DCFH-DA (G) and MitoSOX (H) in BMDMs after different treatments (n = 3). (I) Representative flow cytometry zebra plots of proportion of viable (Q4, Annexin V^−^, PI^−^), early phase apoptotic (Q3, Annexin V^+^, PI^−^), and late phase apoptotic (Q2, Annexin V^+^, PI^+^) BMDMs after different treatments. (J) Quantitative analysis of total apoptosis of BMDMs after different treatments (n = 3). (K) Representative TEM images of mitochondria in BMDMs after different treatments. (L) Scheme illustrating that MQ@Mito preserved mitochondrial homeostasis and attenuated oxidative injury. Data are presented as mean ± SD; ns, not significant; *p < 0.05, **p < 0.01, ***p < 0.001.

Having established the metabolic reprogramming effects, we next examined whether MQ@Mito could maintain mitochondrial homeostasis of macrophages and protect against oxidative stress-induced damage. First, we assessed ΔΨm via JC-1 staining. Flow cytometry showed that TBHP significantly reduced the JC-1 red/green ratio, indicating severe depolarization. Although treatment with MQ or mitochondria alone partially restored ΔΨm, MQ@Mito produced the most robust improvement, confirmed by fluorescence (Fig. 6A and B). Next, we measured intracellular ROS (cROS) and mitochondrial superoxide (mtROS) using DCFH-DA and MitoSOX fluorescent probes. TBHP dramatically increased ROS and superoxide levels. Although MQ or mitochondria monotherapy partially attenuated oxidative stress, MQ@Mito exerted the strongest scavenging effect, significantly reducing both cROS and mtROS (Fig. 6C–H).

Sustained oxidative stress characteristically culminates in programmed cell death [34]. We analyzed apoptosis using Annexin V/PI double staining; total apoptosis was defined as the sum of early apoptotic (Annexin V^+^/PI^−^) and late apoptotic (Annexin V^+^/PI^+^) populations. Flow cytometry revealed that TBHP exposure induced substantial apoptosis in BMDMs, with a total apoptotic rate of 42.43% ± 6.39% compared with 7.66% ± 1.57% in the control group. MQ@Mito treatment significantly rescued this apoptotic cascade, reducing the total apoptosis rate to 15.96% ± 2.13%, notably lower than either MQ or mitochondria alone (Fig. 6I and J). TEM further confirmed that TBHP caused mitochondrial swelling and cristae disruption, while MQ@Mito substantially preserved normal mitochondrial ultrastructure (Fig. 6K). Collectively, these results demonstrated that MQ@Mito mitigated TBHP-induced oxidative stress, restored mitochondrial function, and inhibited apoptosis (Fig. 6L).

Given the above favorable characteristics, we next investigated the protective effects of MQ@Mito on cardiomyocytes. First, CCK-8 assays and live/dead staining confirmed the excellent biocompatibility of MQ in H9c2 cardiomyocytes (Fig. S21). Fluorescence imaging further demonstrated that cardiomyocytes could internalize exogenous mitochondria, and this uptake process was significantly enhanced by MQ (Fig. S22). Notably, MQ@Mito treatment markedly rescued the viability of TBHP-challenged H9c2 cardiomyocytes, confirming its potent cardioprotective effects (Fig. S23). Collectively, these findings demonstrated that MQ@Mito exerted robust cellular protection by maintaining mitochondrial homeostasis, preserving ΔΨm, reducing mitochondrial damage, scavenging both cROS and mtROS, and attenuating oxidative stress-induced apoptosis. Consistently, MQ@Mito protected cardiomyocytes against oxidative stress injury.

### 2.6 MQ^gel^@Mito improved cardiac function in a rat MI model

MQ^gel^ was designed to fabricate a mitochondrial protection and delivery platform (MQ^gel^@Mito). Following confirmation of protective effect *in vitro*, we next verified the absorption period of MQ^gel^@Mito *in vivo*. FITC-labeled MQ^gel^-encapsulated donor mitochondria were injected into the cardiac infarct border zone following left anterior descending (LAD) coronary artery ligation in rats. Fluorescence intensity decreased gradually, with 89.28% ± 2.62% of the system absorbed by day 5 (Fig. S24). This gradual absorption profile enabled sustained release of mitochondria rather than burst release, optimizing organelle utilization while protecting transplanted mitochondria from the harsh post-infarction microenvironment during the critical early period.

**Fig. 7:**
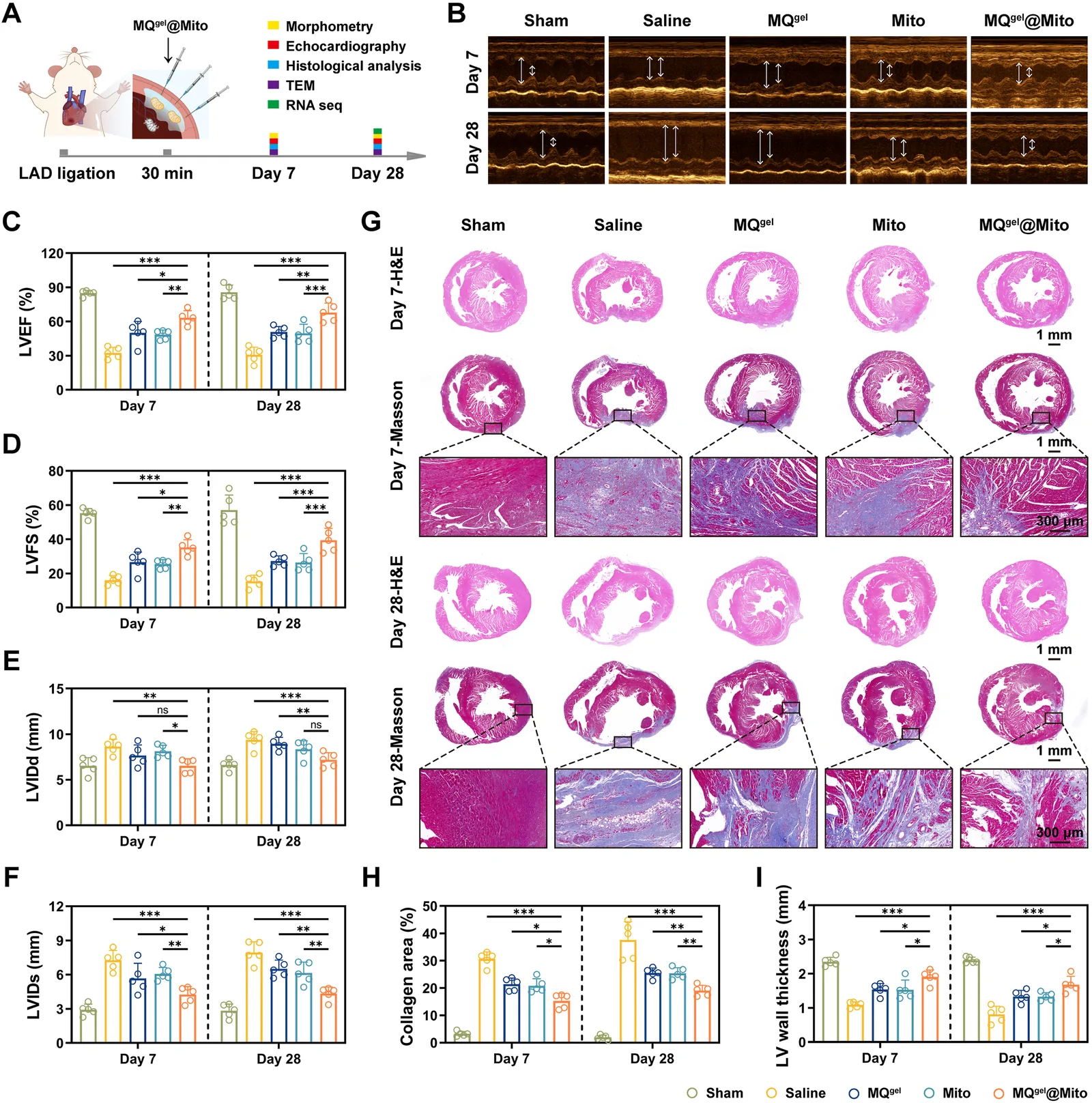
MQ^gel^@Mito improves cardiac function in a rat MI model. (A) Schematic illustration of animal experiments. (B) Representative images of M model echocardiography of different treatments on day 7 and day 28 post-MI (n = 5). The longer line indicates the LVIDd; the shorter line indicates the LVIDs. (C-F) Quantification of LVEF, LVFS, LVIDd, and LVIDs on day 7 and day 28 post-MI (n = 5). (G) Representative images of H&E and Masson's trichrome staining of the rat hearts on day 7 and day 28 post-MI (n = 5). (H-I) Quantification of cardiac collagen area (H) and LV wall thickness (I) on day 7 and day 28 post-MI (n = 5). Data are presented as mean ± SD; ns, not significant; *p < 0.05, **p < 0.01, ***p < 0.001.

We next evaluated the therapeutic efficacy of MQ^gel^@Mito in a rat acute MI model (Fig. 7A). Following permanent LAD ligation, rats received intramyocardial injections of saline, MQ^gel^, mitochondria (Mito), or MQ^gel^@Mito within the infarct border zone. Echocardiographic assessment was performed on day 7 and day 28 post-MI to evaluate cardiac function in rats (Fig. 7B). We first quantified left ventricular ejection fraction (LVEF) across all experimental groups and time points (Fig. 7C). On day 7, the Sham group exhibited normal LVEF (84.92%±2.48%), whereas saline-treated MI rats displayed severe systolic dysfunction (32.48%±4.92%). Separate treatment with MQ^gel^ (50.06%±9.97%) or Mito (48.41%±4.14%) yielded limited functional recovery. In contrast, the MQ^gel^@Mito group achieved a markedly higher LVEF of 63.22%±6.24%, which was superior to all other MI cohorts. On day 28, the intergroup functional hierarchy remained consistent. In the Saline group, LVEF remained low at 30.87%±6.70%; by contrast, monotherapy with MQ^gel^ or Mito yielded only moderate recovery, raising LVEF to 50.84%±4.80% and 49.60%±7.95%, respectively. The combined MQ^gel^@Mito therapy exerted the most potent cardioprotective effect, with the LVEF reaching 67.98%±8.28%. Longitudinal trend analysis showed gradual LVEF deterioration in the Saline group, whereas MQ^gel^@Mito treatment drove sustained, progressive cardiac functional improvement throughout the observational period (Fig. S25A). Left ventricular fractional shortening (LVFS) recapitulated the identical intergroup ranking observed for LVEF on day 7 and day 28 (Fig. 7D). On day 7, MQ^gel^@Mito treatment produced the highest LVFS (35.24%±4.55%) among all MI groups, far outperforming the Saline, MQ^gel^, and Mito cohorts. On day 28, the MQ^gel^@Mito group further differed from single-agent treatments, attaining an LVFS of 39.42%±6.89%, while separate treatment with MQ^gel^ or Mito generated negligible LVFS improvements between the two time points. Longitudinal comparison confirmed that progressive LVFS enhancement was uniquely detected in the MQ^gel^@Mito group (Fig. S25B). Quantitative analysis of left ventricular chamber dimensions—including left ventricular internal diameter in diastole (LVIDd), left ventricular internal diameter in systole (LVIDs), left ventricular end-diastolic volume (LVEDV), and left ventricular end-systolic volume (LVESV)—further corroborated the outstanding anti-remodeling capacity of MQ^gel^@Mito. Post-MI saline-treated rats developed severe ventricular dilation relative to Sham controls, reflecting maladaptive remodeling and progression toward dilated cardiomyopathy. At both time points, MQ^gel^@Mito significantly attenuated ventricular dilation (Fig. 7E and F; Fig. S25C-H).

The rat hearts were harvested on day 7 and day 28 for a series of investigations. Histological analysis using H&E and Masson's trichrome staining corroborated the findings detected by echocardiography. Saline-treated hearts displayed severe fibrosis with substantially increased collagen area (30.60% ± 2.59% on day 7, 37.58% ± 6.66% on day 28) and marked LV wall thinning (1.09 ± 0.08 mm on day 7, 0.81 ± 0.24 mm on day 28). All treatments reduced fibrosis and preserved wall thickness, but MQ^gel^@Mito demonstrated the most pronounced effects, with the lowest collagen area (19.03% ± 1.94%) and thickest LV wall (1.68 ± 0.24 mm) on day 28 (Fig. 7G–I). Safety assessment by histological staining revealed no tissue damage or inflammation in liver, spleen, lung, or kidney tissues (Fig. S26). Blood biochemical analysis revealed that serum levels of alanine aminotransferase (ALT), aspartate aminotransferase (AST), creatinine (Cr), and urea remained within normal physiological ranges in MQ^gel^@Mito-treated rats, with no significant differences compared to the Sham group (Fig. S27), indicating the absence of hepatic or renal toxicity. Overall, these results confirmed that MQ^gel^@Mito facilitated cardiac function and attenuated the adverse remodeling following MI without influencing other organs.

### 2.7 MQ^gel^@Mito attenuated inflammation and facilitated cardiac repair following MI

**Fig. 8:**
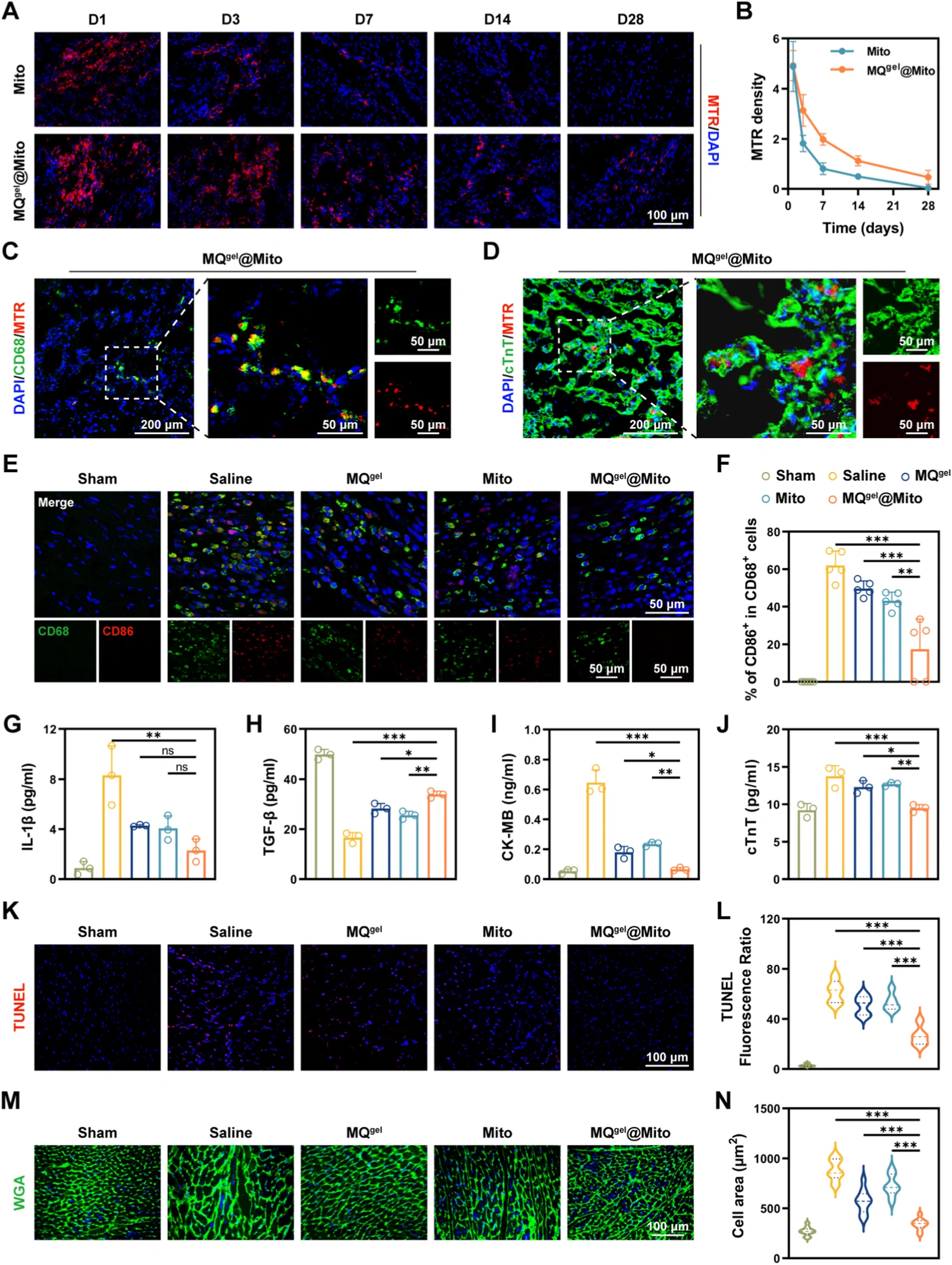
MQ^gel^@Mito attenuates inflammation and facilitates cardiac repair after MI. (A) Representative fluorescence images of MTR-labeled donor mitochondria with or without MQ^gel^ (Mito vs MQ^gel^@Mito) in cardiac tissue at different time points. (B) Quantitative analysis of MTR density in cardiac tissue at different time points (n = 3). (C) Representative fluorescence images of colocalization of MTR-labeled donor mitochondria and macrophage marker (CD68) on day 7 post-MI. (D) Representative fluorescence images of colocalization of MTR-labeled donor mitochondria and cardiomyocyte marker (cTnT) on day 7 post-MI. cTnT, cardiac troponin T. (E) Representative images of CD86/CD68 immunofluorescence staining on day 7 post-MI. (F) Quantitative analysis of the percentage of CD68^+^CD86^+^ pro-inflammatory (M1) macrophages of total CD68^+^ macrophages (n = 5). (G-H) Concentrations of IL-1β (G) and TGF-β (H) in the infarct border zone on day 7 post-MI (n = 3). (I-J) Serum concentrations of CK-MB (I) and cTnT (J) on day 28 post-MI (n = 3). CK-MB, creatine kinase MB. (K) Representative images of TUNEL staining of the infarct border zone of rat myocardium. (L) Quantitative analysis of TUNEL fluorescence ratio in cardiac tissue (n = 5). (M) Representative images of WGA staining. (N) Quantitative analysis of myocyte size (n = 10). Data are presented as mean ± SD; ns, not significant; *p < 0.05, **p < 0.01, ***p < 0.001.

Tracking studies using MTR-labeled donor mitochondria demonstrated that the fluorescence of transplanted mitochondria gradually diminished over time. Donor mitochondria protected by MQ^gel^ exhibited slower fluorescence quenching, with MTR fluorescence still detectable on day 28 post-transplantation. In contrast, donor mitochondria without MQ^gel^ protection, once transplanted into cardiac tissue, showed a significantly faster rate of fluorescence quenching. These findings indicated that MQ^gel^ could prolong the in-situ retention of mitochondria following *in vivo* transplantation (Fig. 8A and B). Immunofluorescence images revealed the colocalization of transplanted mitochondria with CD68^+^ macrophages and cardiac troponin (cTnT)^+^ cardiomyocytes, respectively, confirming internalization by these two cell types (Fig. 8C and D).

Given the anti-inflammatory effects observed *in vitro*, we examined macrophage polarization in the infarct border zone. Immunofluorescence analysis revealed substantial infiltration of CD68^+^CD86^+^ pro-inflammatory (M1) macrophages following LAD ligation. Among total CD68^+^ macrophages, M1 percentage reached 62.02% ± 7.66% in the Saline group on day 7 post-MI. MQ^gel^@Mito dramatically reduced this proportion to 17.38% ± 16.10%, substantially lower than MQ^gel^ (49.67% ± 4.02%) or mitochondria alone (43.17% ± 4.50%) (Fig. 8E and F). Cytokines in the infarct border zone confirmed these findings: pro-inflammatory cytokines IL-1β and TNF-α were markedly elevated at day 7, while anti-inflammatory TGF-β was reduced relative to the Sham group. MQ^gel^@Mito exhibited the most pronounced anti-inflammatory effect and promoted myocardial repair (Fig. 8G and H; Fig. S28).

Subsequently, myocardial injury markers CK-MB and cTnT were significantly reduced in MQ^gel^@Mito-treated animals compared to the Saline group (Fig. 8I and J). TUNEL staining revealed extensive apoptosis in the border zone of infarcted myocardium, consistent with hypoxia and energy depletion. MQ^gel^@Mito substantially reduced TUNEL-positive cells (Fig. 8K and L). Furthermore, wheat germ agglutinin (WGA) staining demonstrated that cardiomyocyte cross-sectional area increased dramatically following MI (883.20 ± 106.30 μm^2^), representing more than 3.3-fold hypertrophy compared to the Sham group, indicating maladaptive compensatory remodeling. MQ^gel^@Mito significantly attenuated this pathological hypertrophy, reducing cardiomyocyte area to 340.10 ± 57.65 μm^2^ (Fig. 8M and N). TEM imaging of the infarct border zone revealed severely impaired mitochondrial ultrastructure in saline-treated animals, characterized by swelling, disrupted cristae, and architectural disorganization. All treatments partially rescued cristae organization, with MQ^gel^@Mito displaying the most well-preserved mitochondrial morphology (Fig. S29). Taken together, these findings demonstrated that MQ^gel^@Mito facilitated cardiac repair by sustaining mitochondrial homeostasis, promoting inflammation resolution, and protecting cardiomyocytes from hypoxic injury.

### 2.8 Transcriptomic analysis confirmed metabolic regulation and anti-inflammatory effects

**Fig. 9:**
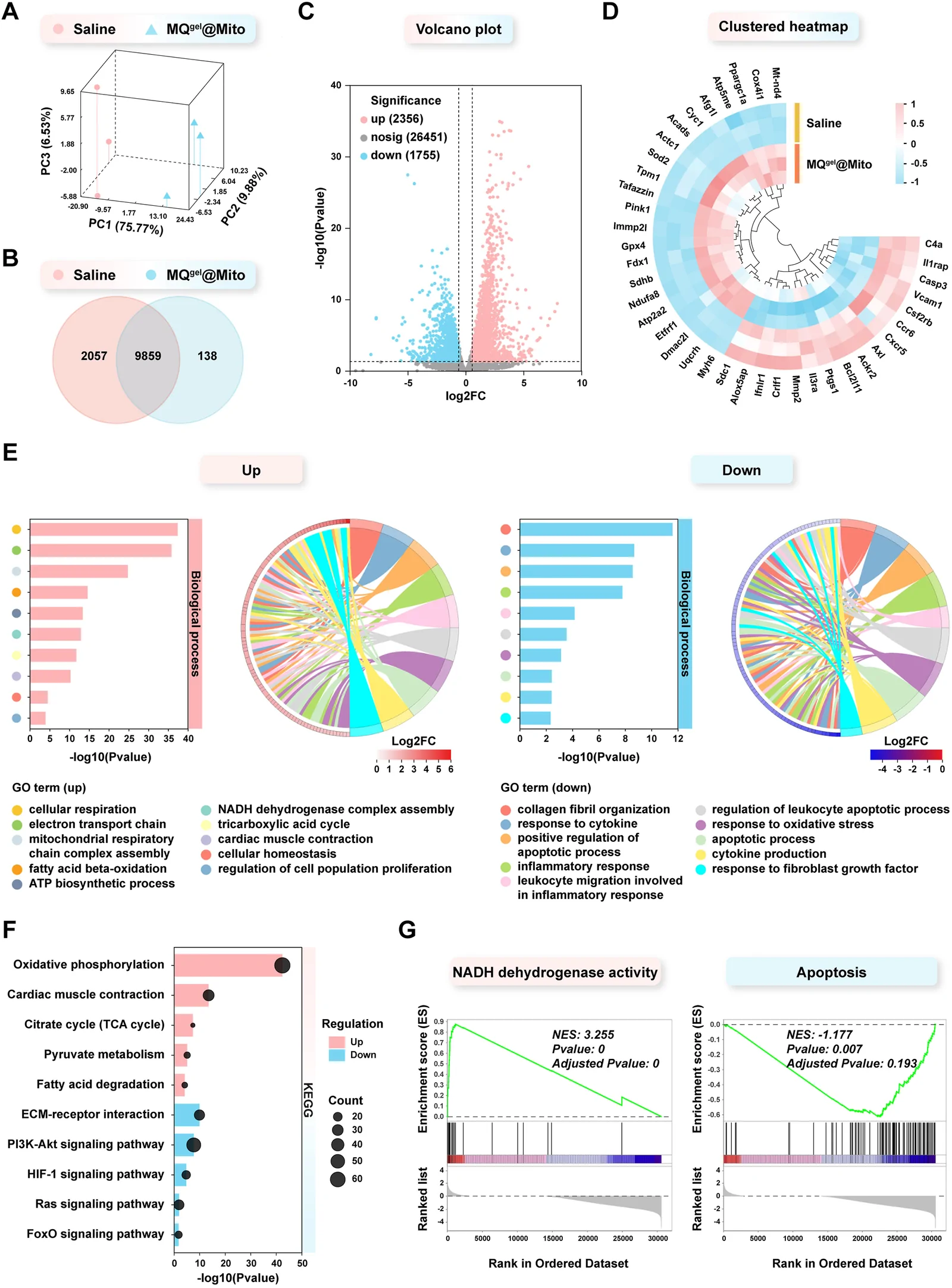
Transcriptomic analysis confirms the metabolic and anti-inflammatory effects of MQ^gel^@Mito. (A) Three-dimensional PCA plot (n = 3). PCA, principal component analysis. (B) Venn diagram (MQ^gel^@Mito vs Saline). (C) Volcano plot of DEGs (MQ^gel^@Mito vs Saline). The pink dots indicate to upregulated DEGs, and the blue dots indicate to downregulated DEGs. |log2FC | > 0.585, *p* value < 0.05. DEGs, differentially expressed genes; FC, fold change. (D) Clustered heatmap of the expression levels of DEGs associated with inflammation, energy metabolism, myocardial contraction, and apoptosis (MQ^gel^@Mito vs Saline). *p* value < 0.05. (E) GO enrichment analysis of up- and downregulated DEGs in biological process categories (MQ^gel^@Mito vs Saline). *p* value < 0.05. GO, gene ontology. (F) KEGG pathway enrichment analysis of up- and downregulated DEGs (MQ^gel^@Mito vs Saline). *p* value < 0.05. (G) GSEA plots showing the enrichment of gene sets associated with NADH dehydrogenase activity and apoptosis (MQ^gel^@Mito vs Saline). GSEA, gene set enrichment analysis; NES, normalized enrichment score. *p* value < 0.05; adjusted *p* value < 0.25; |NES| > 1.

To gain insight into the molecular mechanisms underlying MQ^gel^@Mito therapeutic efficacy, we performed bulk RNA sequencing (RNA-seq) on cardiac tissue from saline- or MQ^gel^@Mito-treated MI rats. The three-dimensional principal component analysis (PCA) revealed clear separation between the two groups, with PC1 accounting for 75.77% of transcriptomic variance, indicating substantial global changes in gene expression (Fig. 9A). The Venn diagram delineated the unique and common expressed genes between the MQ^gel^@Mito and Saline groups, among which 9859 genes were shared (Fig. 9B). DESeq2 was used to analyze differentially expressed genes (DEGs) (MQ^gel^@Mito vs Saline), among which a total of 2356 upregulated and 1755 downregulated genes were found (Fig. 9C).

Analysis of functionally relevant DEGs revealed patterns consistent with our mechanistic studies: genes associated with inflammation (*Il3ra*, *Il1rap*, *Crlf1*) and apoptosis (*Casp3*, *Bcl2l11*) were significantly downregulated, while genes associated with energy metabolism (*Mt-nd4*, *Cox4i1*, *Ndufa8*, *Atp5me*) and myocardial contraction (*Tpm1*, *Actc1*, *Myh6*) were significantly upregulated in the MQ^gel^@Mito group (Fig. 9D). Gene ontology (GO) enrichment analysis identified 10 biological processes that were either upregulated or downregulated following MQ^gel^@Mito treatment (Fig. 9E). Specifically, biological processes associated with mitochondrial respiration and ATP synthesis were upregulated, whereas those related to fibrillation, inflammatory response, and apoptosis were downregulated. Fig. 9F showed 5 upregulated KEGG pathways (pink) and 5 downregulated KEGG pathways (blue), which were consistent with and corroborated the results of the GO enrichment analysis. Among the KEGG pathways, oxidative phosphorylation and the TCA cycle were significantly upregulated. Downregulation of the HIF-1 pathway was closely associated with improved cellular metabolism and inhibited immune-inflammatory responses, while downregulation of the PI3K-AKT pathway was related to reduced cellular stress and alleviated pathological cardiac remodeling. Gene set enrichment analysis (GSEA) further demonstrated that MQ^gel^@Mito upregulated NADH dehydrogenase activity while downregulating apoptosis-related gene sets (Fig. 9G). To validate the transcriptomic findings, we assessed myocardial ATP production and respiratory chain function in the infarct border zone on day 28 post-MI. Compared with saline, MQ^gel^@Mito significantly enhanced the activities of Complex Ⅰ (NADH dehydrogenase) (Fig. S30A) and Complex Ⅴ (ATP synthase) (Fig. S30B), along with increased tissue ATP levels (Fig. S30C). These functional data are consistent with the upregulation of energy metabolism pathways and Complex Ⅰ-related gene sets identified by GO enrichment analysis (Fig. 9E) and GSEA (Fig. 9G).

## 3 Discussion

The resolution of inflammation and subsequent tissue repair after MI hinge on the pivotal transition of cardiac macrophages toward a reparative phenotype, a cell population indispensable for modulating local immune responses in the heart [35]. The phenotype of macrophages is closely related to the metabolic pathway [36]. MTT is an emerging approach for the treatment of diseases with mitochondrial dysfunction such as MI [31]. However, the clinical translation of MTT is severely constrained by three interrelated bottlenecks: the fragility of mitochondria, the hostile post-MI microenvironment, and the low efficiency of mitochondrial internalization by recipient cells [12,15,16,37]. Here, inspired by the intrinsic cellular defense mechanism against mitochondrial dysfunction, we reported MQ^gel^@Mito, a mitochondria-derived peptide hydrogel system encapsulating functional mitochondria. This system substantially extended organelle viability, enhanced internalization by macrophages, and promoted cardiac repair through macrophage metabolic reprogramming.

Different from conventional mitochondrial delivery systems that rely merely on passive physical protection, the superior protective capacity of MQ^gel^ for mitochondria stems from the combination of physical shielding and intrinsic bioactive effects. Mechanistically, the hydrogel matrix serves as a protective barrier that insulates encapsulated mitochondria from detrimental extracellular microenvironments [17,38]. Additionally, the hydrogel matrix, with its porous three-dimensional architecture and highly hydrated environment, prevents mitochondrial aggregation and maintains mitochondrial dispersion, ensuring efficient exchange of metabolic substrates and byproducts to sustain mitochondrial metabolic activity [18,39]. Beyond passive physical protection, MQ^gel^ integrates the mitochondrial-derived peptide MOTS-c and exhibits inherent bioactivity capable of stabilizing mitochondrial energy metabolism and eliminating excessive ROS [26]. Our data revealed that 30 mg mL^−1^ MQ^gel^ extended this window to 8 h, a remarkable improvement that transformed MTT from a logistically constrained procedure into a clinically tractable intervention. Besides, the donor mitochondria are transplanted to a harsh transplantation environment with excess oxidative stress and calcium overload following MI [17,40]. Our findings demonstrated that MQ^gel^-encapsulated mitochondria exhibited markedly preserved ATP synthesis ability, respiratory chain function, and mitochondrial membrane potential following exposure to these adverse factors. These protective effects are particularly consequential during the critical period between intramyocardial delivery and cellular internalization, when naked mitochondria would otherwise be vulnerable to environmental insult.

Donor mitochondria should be internalized by recipient cells in order to exert their functions [9,34,41]. A central finding of this work is that the MQ enhanced mitochondrial internalization through an AMPK-dependent mechanism. As reported, mitochondria can be internalized via macropinocytosis [16]. As a cellular energy sensor, AMPK pathway can drive cytoskeletal remodeling by activating the actin-severing protein cofilin, thereby enhancing the formation of macropinosome [[27], [28], [29]]. Therefore, our results suggested that MQ facilitated the internalization of isolated mitochondria by macrophages through AMPK-driven macropinocytosis. The observed colocalization of donor mitochondria with the recipient mitochondrial network suggested functional integration rather than lysosomal degradation, which was a critical consideration for sustained therapeutic benefit.

Unlike previous studies focusing on direct cardiomyocyte protection, our work focuses on the immunometabolic effects of MQ@Mito and its contribution to cardiac tissue regeneration post MI. Macrophage polarization is intimately coupled to cellular metabolism: pro-inflammatory macrophages rely upon glycolysis with impaired OXPHOS, whereas pro-reparative macrophages depend upon mitochondrial function [36]. Glycolysis provides a rapid but less efficient ATP yield (∼2 per glucose), whereas an intact TCA cycle maintains a sustained supply of ATP (∼30 per glucose) through OXPHOS and fatty acid oxidation [42]. LPS disrupts the TCA cycle by inhibiting the expression and activity of isocitrate dehydrogenase, thereby causing the accumulation of isocitrate and its upstream metabolite, cis-aconitate [43]. Our metabolomic and functional analyses revealed that by providing functionally intact mitochondria, MQ@Mito treatment suppressed LPS-induced glycolysis while maintaining TCA cycle integrity and enhancing OXPHOS capacity. This metabolic reprogramming was accompanied by phenotypic changes consistent with attenuated pro-inflammatory M1 polarization and enhanced M2 polarization. The mechanistic link between bioenergetic state and inflammatory phenotype underscored the potential of metabolism-targeted interventions for modulating macrophage function (Fig. 5P).

*In vivo*, MQ^gel^@Mito prevented the rapid decline in myocardial contractility following MI, resulting in a marked improvement in cardiac function, inhibited adverse remodeling, and alleviated thinning of LV wall. The efficacy of MQ^gel^@Mito for MI treatment substantially exceeded that of either MQ^gel^ or mitochondrial transplantation alone. MQ^gel^ protected donor mitochondria against the hostile transplantation environment with oxidative stress and calcium overload in the infarct border zone and thereby enhanced the function of donor mitochondria. The controlled degradation profile of MQ^gel^ enabled sustained mitochondrial release rather than burst delivery, optimizing the absorption and utilization of organelles during the critical early post-MI period. Macrophages are one of the most active cell types across all post-MI stages, including the inflammatory, cardioprotective, and cardiac repair phases [4]. Macrophage-targeted therapy plays a pivotal role in post-MI recovery. We found that MQ^gel^@Mito inhibited the infiltration of M1-like macrophages, decreased secretion of pro-inflammatory cytokines, and increased secretion of reparative cytokines, thereby significantly mitigating excessive inflammatory response after MI. The mechanism of anti-inflammation was closely associated with the metabolic reprogramming of macrophages induced by MQ^gel^@Mito. Our transcriptomic analysis provided molecular confirmation of the observed functional effects. In MQ^gel^@Mito-treated rat hearts, the upregulation of genes encoding respiratory chain components (*Mt-nd4*, *Cox4i1*, *Ndufa8*, *Atp5me*) and contractile proteins (*Tpm1*, *Actc1*, *Myh6*), coupled with downregulation of inflammatory (*Il3ra*, *Il1rap*) and apoptotic (*Casp3*, *Bcl2l11*) genes, aligned with the improved energy metabolism, enhanced contractility, attenuated inflammation, and reduced cell death.

Despite promising results, several limitations exist in the current study. First, although we demonstrated that MQ^gel^ extended mitochondrial viability, the optimal storage duration and conditions for clinical implementation require further systematic optimization. Second, the source of donor mitochondria, autologous versus allogeneic, remains to be addressed. While allogeneic mitochondria could enable off-the-shelf therapy, potential immune considerations warrant investigation. Third, MTR labeling, although widely employed for *in vivo* mitochondrial tracking, has inherent limitations: because the dye covalently binds mitochondrial proteins, residual fluorescence from degraded organelles may overestimate the presence of functional mitochondria [44]. Advanced technologies capable of reliably distinguishing exogenous from endogenous mitochondria post-transplantation are needed to fully elucidate the *in vivo* fate, distribution, and integration of transplanted mitochondria. Finally, to further dissect the therapeutic mechanism of MTT in post-MI treatment, macrophage-specific depletion models, single-cell sequencing, and lineage-tracing approaches are warranted to delineate cell-type-specific contributions and resolve the relative importance of macrophage-mediated immunomodulation.

## 4 Conclusion

In summary, we successfully developed MQ^gel^@Mito, a robust platform for mitochondrial protection and transplantation that overcame the fundamental viability constraints limiting current MTT approaches. *In vitro*, MQ^gel^ protected donor mitochondria from oxidative stress and calcium overload in the transplantation environment, facilitating the survival and function of mitochondria. MQ promoted the internalization of donor mitochondria by macrophages via an AMPK-dependent macropinocytic mechanism. Furthermore, MQ@Mito modulated the polarization of macrophages by metabolic reprogramming and attenuated apoptosis by maintenance of mitochondrial homeostasis and weakened oxidative stress. *In vivo*, we confirmed that MQ^gel^@Mito improved the efficacy of MTT, markedly improving cardiac function and inhibiting the pathological adverse remodeling post-MI. By supplying more functional donor mitochondria to the infarct border zone and exerting the metabolic effect of MQ^gel^, MQ^gel^@Mito achieved therapeutic effects substantially exceeding those of either component alone with the weakest inflammatory response and cardiac damage. The demonstration that macrophage immunometabolism can be therapeutically targeted to promote cardiac repair opens new avenues for regenerative medicine beyond the cardiovascular system. As mitochondrial dysfunction underlies diverse pathologies, including neurodegenerative diseases, metabolic disorders, and inflammatory conditions, the principles established here may find broad application across therapeutic domains. This integrated platform substantially improves cardiac function and limits pathological remodeling in rats, highlighting the therapeutic potential of targeting macrophage immunometabolism in regenerative medicine. Given that mitochondrial dysfunction underlies many diseases, the principles established here may extend beyond cardiovascular repair.

## 5 Methods

### 5.1 Mitochondrial isolation

Mitochondria were isolated from skeletal muscle tissues of SD rats using the Tissue Mitochondria Isolation Kit (Cat. No. C3606, Beyotime). Fresh muscle tissues were rinsed with ice-cold PBS to remove contaminants and minced into small pieces. The minced tissues were digested with pre-cooled trypsin and incubated on ice for 20 min. After brief centrifugation to collect tissue pellets, samples were homogenized on ice in pre-chilled Mitochondrial Isolation Reagent B (800 μL per 100 mg tissue) with 20 gentle strokes using a Dounce homogenizer. The homogenate was centrifuged at 1000 *g* for 5 min at 4 °C, and the resulting supernatant was further centrifuged at 3500 *g* for 10 min at 4 °C. The final sediment was collected as purified mitochondria and resuspended in mitochondrial respiratory substrate solution containing 10 mM glutamate, 5 mM malate, and 10 mM ADP.

### 5.2 MQ^gel^@Mito preparation

MQ^gel^ was prepared by dissolving MQ peptide in double-distilled water (ddH_2_O) to achieve final concentrations of 20, 30, and 40 mg mL^−1^, followed by the addition of an appropriate volume of saturated NaCl solution and immediate shaking at room temperature. For fabrication of the MQ^gel^@Mito composite system, the isolated mitochondrial sediment was first resuspended in a moderate volume of respiratory substrate solution and then thoroughly mixed with pre-formed MQ^gel^.

### 5.3 Isolation and cultivation of BMDMs

BMDMs were isolated from 6-week-old female C57BL/6J mice. Briefly, femurs and tibiae were aseptically dissected, and bone marrow was flushed with ice-cold PBS using a sterile syringe. The resulting cell suspension was filtered through a 70-μm cell strainer to remove bone fragments and tissue debris. Cells were pelleted by centrifugation at 800*g* for 8 min at 4 °C and resuspended in erythrocyte lysis buffer (Cat. No. C3702, Beyotime). Following 2-min lysis, the reaction was terminated with ice-cold PBS. Cells were then centrifuged at 450 *g* for 8 min at 4 °C and resuspended in RPMI-1640 medium (Cat. No. C11875500BT, Gibco) supplemented with 10% heat-inactivated fetal bovine serum (FBS, inactivated at 56 °C for 30 min; Cat. No. 164210, Procell), 1% penicillin-streptomycin solution (Cat. No. P1400, Solarbio), and 20 ng mL^−1^ recombinant macrophage colony-stimulating factor (M-CSF, Cat. No. HY-P7085, MCE). Cells were seeded in culture dishes and incubated at 37 °C in a humidified atmosphere containing 5% CO_2_. On day 3, half of the culture medium was replaced with an equal volume of fresh complete medium containing M-CSF. After 6 days of differentiation, adherent mature macrophages were harvested for subsequent experiments.

### 5.4 Internalization of mitochondria by BMDMs

To investigate the effect of different concentrations of MQ peptide on mitochondrial uptake by macrophages, BMDMs were isolated and cultured in 6-well plates at a density of 1 × 10^6^ cells per well until day 6. The cells were divided into three groups and treated with MQ peptide at concentrations of 0, 10, and 25 μM, respectively. After 24 h of treatment, MTR (Cat. No. C1032, Beyotime)-labeled isolated mitochondria were co-incubated with BMDMs at a dose of 1 × 10^6^ mitochondrial particles per well. At 2, 4, 8, and 12 h post-incubation, BMDMs were harvested and washed three times with PBS. The MFI (FL4-A) of BMDMs was quantified by flow cytometry using a flow cytometer (C6 Plus, BD Biosciences, San Jose, CA, USA) to evaluate the number of internalized mitochondria. To visually characterize the cell uptake of mitochondria, the BMDMs were stained by MTG (Cat. No. C1048, Beyotime) to label the endogenous mitochondria and then incubated with MTR-labeled exogenous mitochondria (1 × 10^6^ particles per confocal dish) for 2 and 12 h. The fluorescent images were acquired using a laser scanning confocal microscope (TCS SP5II, Leica, Germany).

To investigate the effect and underlying mechanism of MQ on mitochondrial uptake by macrophages, BMDMs were divided into three groups: mitochondria (Mito), 25 μM MQ with mitochondria (MQ@Mito), and MQ@mito+si-*AMPKα1*. MTR-labeled mitochondria (3 × 10^5^ particles per well) were added for co-incubation with BMDMs. Other procedures were performed as described above.

To investigate the effect and underlying mechanism of MQ on mitochondrial uptake by M1-polarized macrophages, BMDMs were induced into M1-like macrophages by stimulation with 200 ng mL^−1^ LPS (Cat. No. L8880, Solarbio) for 48 h and then divided into three groups: Mito, MQ@Mito, and MQ@Mito+si-*AMPKα1*. Other procedures were performed as described above.

### 5.5 Western blot

Total proteins were extracted from purified mitochondria or BMDMs using RIPA lysis buffer (Cat. No. P0013B, Beyotime) supplemented with protease and phosphatase inhibitors (Cat. No. P1045, Beyotime). Proteins were separated by SDS-PAGE and electrotransferred onto 0.22-μm-thick PVDF membranes. Membranes were blocked with 5% bovine serum albumin (BSA) for 1 h at room temperature, and then incubated overnight at 4 °C with primary antibodies. All Western blot primary antibodies involved in the entire study are listed as follows: COXIV (Cat. No. ab202554, Abcam), β-tubulin (Cat. No. LF203S, Epizyme), AMPK (Cat. No. 2532, CST), p-AMPK (Cat. No. 2535, CST), β-actin (Cat. No. 20536-1-AP, Proteintech). After six TBST washes, membranes were incubated with HRP-conjugated anti-rabbit or anti-mouse IgG (H + L) secondary antibodies (Cat. No. AS014, Abclonal; Cat. No. 7076, CST) for 1 h at room temperature. Following six TBST washes, protein bands were visualized using an enhanced chemiluminescence (ECL) detection system (Cat. No. P10100, NCM Biotech). Band intensities were densitometrically analyzed using ImageJ software with β-actin or β-tubulin as internal references.

### 5.6 Flow cytometric analysis of macrophage polarization

BMDMs were polarized into M1 macrophages via stimulation with 200 ng mL^−1^ LPS for 48 h. To evaluate the effects of MQ@Mito on macrophage polarization, MQ peptide, isolated mitochondria, or MQ@Mito were administered to BMDMs at three different time points: 24 h prior to LPS exposure, simultaneously with LPS stimulation, or 24 h after LPS treatment. Subsequently, cells were harvested and co-stained with PE anti-mouse F4/80 antibody (Cat. No. 123110, BioLegend) and FITC anti-mouse CD86 antibody (Cat. No. 105005, BioLegend) at room temperature for 40 min in the dark. After three washes with PBS, BMDMs were resuspended in 4% paraformaldehyde (PFA, Cat. No. P1110, Solarbio) fixative and analyzed by flow cytometry. The proportion of M1 macrophages was quantified as the percentage of F4/80^+^CD86^+^ BMDMs using appropriate gating strategies with FlowJo software v10.8.1.

For M2 polarization induction, BMDMs were treated with MQ peptide, isolated mitochondria, or MQ@Mito for 48 h, with 40 ng/mL IL-4 treatment serving as the positive control. After treatment, BMDMs were harvested and stained with F4/80 antibody. Subsequently, the cells were fixed and permeabilized with 0.1% Triton X-100, followed by incubation with APC anti-mouse CD206 antibody (Cat. No. 141708, BioLegend) for 40 min in the dark. All other experimental procedures were consistent with those used for M1 polarization. The proportion of M2 macrophages was quantified as the percentage of F4/80^+^CD206^+^ BMDMs.

### 5.7 Metabolic profiling analysis

For targeted energy metabolomics analysis, BMDMs subjected to different treatments (control, LPS stimulation, LPS + MQ^gel^@Mito intervention) were collected. Cells were washed twice with ice-cold PBS to remove residual medium and debris, and cellular metabolism was rapidly quenched by pre-chilled extraction solvent before cell lysis to preserve endogenous metabolites. Targeted energy metabolomics profiling was performed using liquid chromatography-tandem mass spectrometry (LC-MS/MS) by Metware Biotechnology Co., Ltd. (Wuhan, China). Three independent biological replicates were included for each group, with each sample containing **>**1 × 10^6^ BMDMs to ensure sufficient metabolite abundance and detection reliability. Raw data were processed for peak identification, metabolite annotation, and quantitative normalization. Metabolic profiling, multivariate statistical analysis, and data visualization were performed using online analytical platforms: Metware Cloud Platform (https://cloud.metware.cn/) and CNSknowall (https://www.cnsknowall.com/).

### 5.8 Animal models

All animal procedures were performed in accordance with the Guidelines for the Care and Use of Laboratory Animals of Peking Union Medical College and approved by Institutional Animal Care and Use Committee (IACUC), Fuwai Hospital, Chinese Academy of Medical Sciences (IACUC Issue No.: 0109-7-1359-ZX(X)-30).

Male Sprague Dawley rats (Vital River Laboratory, Beijing, China) weighing 200 ± 20 g at 6 weeks of age were used to establish MI models. Anesthesia was induced via intramuscular injection of a mixed anesthetic agent containing Zoletil 50, xylazine hydrochloride injection, and normal saline at a volume ratio of 1:1:8, at a dose of 0.11 mL per 100 g body weight. Subsequently, endotracheal intubation was performed with a 16-G trocar, and mechanical ventilation was maintained with a ventilator. Thoracotomies were performed at the fourth intercostal space to expose the heart. Rats were randomly assigned to five groups: Sham, Saline, MQ^gel^, Mito, and MQ^gel^@Mito groups. The LAD coronary artery was ligated with a 6-0 nylon suture. The pallor of the cardiac apex indicated the successful establishment of MI model. Following 30 min of ischemia, 100 μL saline, MQ^gel^ (30 mg mL^−1^), Mito (mitochondrial suspension), or MQ^gel^@Mito was injected into the infarct border zone in corresponding groups. The sham group underwent identical surgical procedures without LAD ligation or injection. Then, the thoracic cavity was carefully closed with 3-0 nylon sutures, followed by skin closure using the same suture material. Postoperatively, rats were placed in a temperature-controlled incubator for rewarming, and respiratory rhythm was monitored until full recovery from anesthesia. All rats were then housed in standard breeding cages. Intramuscular penicillin injection was administered for three consecutive days for postoperative anti-infection prophylaxis.

### 5.9 Echocardiography

Rats were anesthetized with the aforementioned combined anesthetic agent, and standard transthoracic echocardiography was performed using an ultrasound imaging system on day 7 and 28 post-treatment. Cardiac functional parameters including LVEF, LVFS, LVIDD, LVIDS, LVEDV, and LVESV were acquired and analyzed.

### 5.10 Immunofluorescence analysis

Rats were euthanized, and cardiac tissues were harvested, fixed in 4% PFA, paraffin-embedded, and sliced into 4-μm-thick sections. After baking at 60 °C for 1 h, dewaxing, rehydration, and antigen retrieval using citrate antigen retrieval powder (Cat No. E673002, Sangon Biotech), sections were permeabilized with 0.1% Triton X-100 (Cat. No. P0096, Beyotime) and blocked with goat serum. Subsequently, the sections were incubated overnight at 4 °C in the dark with primary antibodies. All immunofluorescent primary antibodies involved in the entire study are listed as follows: CD68 (Cat. No. ab283654, Abcam), CD86 (Cat. No. ab220188, Abcam), cTnT (Cat. No. ab209813, Abcam). Following three washes with PBS, sections were incubated for 2 h at room temperature in the dark with HRP-conjugated goat anti-rabbit or anti-mouse IgG (H + L) secondary antibodies (Cat. No. A11008, Invitrogen; Cat. No. ab175473, Abcam). After another three PBS washes, sections were mounted with DAPI-containing antifade mounting medium (Cat. No. ab104139, Abcam). Immunofluorescent images were acquired using a laser scanning confocal microscope.

### 5.11 Bulk RNA-seq

Cardiac tissues from rats in the Saline group and the MQ^gel^@Mito group were collected for bulk RNA-seq. Library construction and transcriptome sequencing were performed by Majorbio Bio-Pharm Technology Co., Ltd. (Shanghai, China). Briefly, total RNA was extracted and purified from tissue samples, followed by quality control to assess RNA integrity and purity. Qualified RNA samples were used for cDNA library preparation, and high-throughput sequencing was conducted on an Illumina platform. Raw sequencing reads were filtered, aligned to the rat reference genome, and quantified for gene expression levels. Transcriptomic data analysis, including differential expression analysis, functional enrichment analysis, and data visualization, was performed using Majorbio Cloud Platform (https://www.majorbio.com/tools); and Chiplot online visualization tool (https://www.chiplot.online/).

### 5.12 Statistical analysis

All statistical analyses were performed using GraphPad Prism 9.5 software. Data are presented as mean ± SD. An unpaired Student's t-test was used to compare differences between two independent groups. One-way analysis of variance (ANOVA) followed by Dunnett's multiple comparisons test was utilized to compare each experimental group versus the control group, with statistical significance evaluated for each individual comparison. One-way ANOVA with Tukey's multiple comparisons test was employed for multiple comparisons among all groups. Statistical significance was defined as follows: ns, not significant; *p < 0.05, **p < 0.01, ***p < 0.001.

## Data availability statement

Research data are not shared.

## Ethics approval and consent to participate

All animal procedures were performed in accordance with the Guidelines for the Care and Use of Laboratory Animals of Peking Union Medical College and approved by Institutional Animal Care and Use Committee (IACUC), Fuwai Hospital, Chinese Academy of Medical Sciences (IACUC Issue No.: 0109-7-1359-ZX(X)-30).

## CRediT authorship contribution statement

**Hang Li:** Conceptualization, Data curation, Formal analysis, Investigation, Methodology, Validation, Writing – original draft. **Yushan Zhang:** Conceptualization, Data curation, Formal analysis, Investigation, Methodology, Validation, Writing – original draft. **Yifan Jian:** Formal analysis, Investigation, Methodology, Validation. **Fang Fang:** Formal analysis, Supervision, Validation, Writing – review & editing. **Wenbin Ouyang:** Formal analysis, Supervision, Validation, Writing – review & editing. **Donglin Zhuang:** Formal analysis, Supervision, Validation, Writing – review & editing. **Wenhao Ju:** Conceptualization, Project administration, Resources, Supervision, Writing – review & editing. **Rui Gao:** Formal analysis, Investigation, Methodology, Validation. **Yu Gao:** Formal analysis, Investigation, Methodology, Validation. **Shaoyang Kang:** Formal analysis, Investigation, Methodology, Validation. **Pengxu Kong:** Formal analysis, Funding acquisition, Investigation, Methodology, Validation. **Yuwei Li:** Formal analysis, Investigation, Methodology, Validation. **Xiangbin Pan:** Conceptualization, Funding acquisition. **Weiwei Wang:** Conceptualization, Funding acquisition, Project administration, Resources, Supervision, Writing – review & editing. **Zujian Feng:** Conceptualization, Funding acquisition, Project administration, Resources, Supervision, Writing – review & editing.

## Declaration of competing interest

The authors declare no conflict of interest.
