# Supplementary material for: Mitochondria-derived peptide hydrogel augments mitochondrial transplantation for promoting cardiac repair via macrophage metabolic reprogramming
Source: Bioact Mater. 2026 Aug 14;67:458–76. doi: 10.1016/j.bioactmat.2026.06.010 (PMC13499492; doi:10.1016/j.bioactmat.2026.06.010)
Supplement: Supplementary file 1 [file mmc1.docx]

**Supplementary Information**

**Mitochondria‑derived Peptide Hydrogel Augments Mitochondrial Transplantation for** **Promoting Cardiac Repair via Macrophage Metabolic Reprogramming**

**Supporting experimental methods**

*JC-1 assay*: JC-1 working solution was prepared by diluting JC-1 (200X) in JC-1 staining buffer (Cat. No. C2003S, Beyotime) at a ratio of 5 µL JC-1 per 1 mL buffer, followed by thorough mixing via pipetting. To 0.9 mL of the JC-1 working solution, 0.1 mL of purified mitochondria (10-100 µg total protein) were added. After incubation at 37 °C for 20 min, the staining solution was rinsed off with PBS. Fluorescence was immediately measured using a spectrofluorometer or a fluorescence microplate reader in time-scan mode, with excitation and emission wavelengths set at 485 nm and 590 nm, respectively. After fluorescence detection, the red-to-green fluorescence ratio was calculated for each sample.

*ATP assay*: Mitochondria were lysed using ice-cold lysis buffer. The lysate was centrifuged at 12000 *g* for 5 min at 4 ℃. The resulting supernatant was collected for subsequent ATP measurement (Cat. No. S0027, Beyotime). ATP standard solution was thawed on ice and serially diluted with ATP assay lysis buffer to generate a calibration curve ranging from 0.01 to 10 μM. The following concentration points were included: 0.01, 0.03, 0.1, 0.3, 1, 3, and 10 μM. An appropriate volume of ATP assay reagent was diluted 1:4 with ATP assay dilution buffer to prepare the working solution. A total of 100 μL of ATP working solution was aliquoted into each well or tube and incubated at room temperature for 5 minutes to allow degradation of background ATP. Then, 20 μL of sample or standard was added to each well/tube and mixed rapidly using a micropipette. After a delay of at least 2 seconds, luminescence was measured using a luminometer.

*Circular dichroism (CD) spectroscopy*: The secondary structure characteristics of MOTS-c, Q11, and MQ peptide samples were determined using circular dichroism spectroscopy. All samples were prepared with uniform concentration and loaded into a quartz cuvette with a 0.1 cm optical path length. Spectral scanning was performed at a wavelength range of 190-260 nm, with a scanning speed of 50 nm/min and a bandwidth of 1 nm at room temperature. Baseline correction was performed using pure solvent blank to eliminate background interference. Each sample was scanned repeatedly three times to obtain averaged spectra. The final CD spectra were analyzed and fitted to quantify the relative contents of α-helix, β-sheet, random coil, and β-turn structures using the BESTSEL algorithm.

*Morphology assessment*: The ultrastructure of mitochondria was assessed by TEM (JEM-1200EX, Japan) following fixation by 2.5% glutaraldehyde. The microscopic morphology and nanostructure of MOTS-c, Q11, and MQ peptide samples were observed via negative staining TEM. Samples were imaged using a JEM-2100Plus transmission electron microscope (JEOL Ltd., Tokyo, Japan) at an accelerating voltage of 200 kV. The morphology of MQ^gel^ was assessed by SEM (ZEISS Sigma 360, Germany) following rapid freeze of samples in liquid nitrogen and subsequent lyophilization.

*Rheology testing*: The rheological properties of MQ^gel^ and MQ^gel^@Mito were evaluated using an AR 2000 ex rheometer. The angular frequency sweep was measured with a scan frequency from 0.2 to 35 rad s^-1^ and a strain of 1%. The strain sweep was carried out under a scan frequency of 1 rad s^-1^ and a strain from 2% to 101%. The self-healing property was evaluated by measuring the changes in G′ and G′′ under continuous step sweeps at a low strain (1%) and a high strain (100%).

*Assessment of mitochondrial respiratory chain Complex Ⅰ, Complex Ⅲ, Complex Ⅳ, and Complex Ⅴ activity*: Isolated mitochondria with different treatments were collected. Mitochondrial respiratory chain Complex Ⅰ, Ⅲ, and Ⅳ activity was measured using the mitochondrial respiratory chain complex assay kits (Cat. No. AKOP005M, BoxBio; Cat. No. AKOP007M, BoxBio; Cat. No. AKOP008M, BoxBio) according to the standard protocols. The concentration of mitochondrial protein was determined via the BCA assay, and the obtained protein content was used as the normalization standard for the activity detection of mitochondrial respiratory chain complexes. Appropriate tissue samples were collected and weighed from the marginal area of myocardial infarction. The activities of mitochondrial respiratory chain Complex Ⅰ and Complex Ⅴ (Cat. No. AKOP009M, boxbio) were detected in accordance with the manufacturer’s instructions, and the tissue weight was used for data normalization.

*Continuous Z-stack scanning*: For three-dimensional (3D) morphological observation and spatial localization analysis, MQ^gel^@Mito composites were fluorescently labeled with specific probes, with FITC applied for green fluorescence labeling of MQ^gel^ and MTR for red fluorescence labeling of mitochondria. Continuous Z-stack scanning was performed using a laser scanning confocal microscope (TCS SP5II, Leica, Germany) with an appropriate step size, covering the entire vertical thickness of MQ^gel^@Mito. Serial layered fluorescence images were acquired sequentially, and 3D reconstruction was processed. The reconstructed 3D models were applied to intuitively observe the spatial distribution of mitochondria in MQ^gel^.

*Degradation of MQ^gel^@Mito in vitro*: It was evaluated via a gravimetric method. Pre-weighed MQ^gel^@Mito samples were immersed in PBS and incubated at 37 ℃. At predetermined time intervals, the residual hydrogel samples were collected and weighed. The degradation rate was calculated according to the remaining mass fraction of MQ^gel^@Mito over incubation time.

*Mitochondrial release profile of MQ^g^*^el^*@Mito in vitro*: Isolated mitochondria were pre-labeled with MTR and encapsulated in MQ^gel^, followed by incubation in PBS at 37 ℃. The supernatant was harvested at scheduled time points, and the number of released mitochondria in the supernatant was quantitatively analyzed by flow cytometry.

*Cell counting kit-8 (CCK-8) assay:* Cells were seeded into 96-well plates at a uniform density and cultured overnight. After different treatments (Control, 25 μM MQ) for 1 day and 3 days, the culture medium was replaced with fresh medium containing 10% CCK-8 working solution (Cat. No. HY-K0301, MCE). The plates were incubated at 37 ℃ in a 5% CO_2_ incubator for 30 min in the dark. Cell viability was evaluated by detecting absorbance measured at a wavelength of 450 nm using a microplate reader.

*Live/dead cell staining assay*: Cells were seeded into 6-well plates at a uniform density and cultured overnight. After different treatments (Control, 25 μM MQ) for 1 day and 3 days, a working staining solution containing Calcein-AM and PI (Cat. No. C2015S, Beyotime) was diluted at a ratio of 1:1000 and added to cover the cell monolayer. Cells were incubated at 37 ℃ for 30 min in the dark. Following staining, the staining solution was removed, and cells were washed once with PBS. Fluorescence images were immediately captured using an inverted fluorescence microscope, where live cells emitted green fluorescence and dead cells emitted red fluorescence.

*qPCR*: Total RNA of BMDMs was isolated using the total RNA Extraction Kit (Cat. No. R1200, Solarbio). The amount and purity of RNA in each sample were quantified using the NanoDrop ND-1000 (NanoDrop, USA). The total RNA was reverse transcribed into cDNA using a transcription kit (Cat. No. R323-01, Vazyme), and the target genes were amplified using AceQ Universal SYBR qPCR Master Mix (Cat. No. Q712, Vazyme). The sequence (5′-3′) of each pair of primers are listed as follows: *AMPKα1* (mouse):

5'-CCGTAGTATTGATGATGAGA-3' (forward primer)

5'-CGATAGTTGCTGATGGAT-3' (reverse primer)

*AMPKα2* (mouse):

5'-GCTTCATCTGTAGTCTCT-3' (forward primer)

5'-TGTGGTATCTGTGTTAGG-3' (reverse primer).

*Tnf-α* (mouse):

5'-CCCTCACACTCACAAACCAC-3' (forward primer)

5'-ACAAGGTACAACCCATCGGC-3' (reverse primer)

*Arg1* (mouse):

5'-CTCCAAGCCAAAGTCCTTAGAG-3' (forward primer)

5'-AGGAGCTGTCATTAGGGACATC-3' (reverse primer)

*Il10* (mouse):

5'-GCTCTTACTGACTGGCATGAG-3' (forward primer)

5'-CGCAGCTCTAGGAGCATGTG-3' (reverse primer).

*Tgf-β* (mouse):

5'-ACTGGAGTTGTACGGCAGTG-3' (forward primer)

5'-GGGGCTGATCCCGTTGATTT-3' (reverse primer).

*siRNA transfection of BMDMs*: BMDMs were cultured in 24-well plates for 6 days after isolation to allow full differentiation into mature macrophages. On day 6, the cells were transfected with si-*AMPK* or si-NC using an siRNA-Mate SUS Kit (Cat. No. G04035, GenePharma) per the manufacturer’s protocol. A total of 25 pmol siRNA was complexed with siRNA-Mate SUS 2X Buffer and siRNA-Mate SUS Reagent, and then added to BMDMs cultured in complete medium. *AMPK* knockdown efficiency was evaluated by qPCR at 24 h post-transfection and by Western blot analysis at 48 h post-transfection.

*Dextran uptake assay*: For the detection of macrophage endocytic activity, BMDMs were incubated with serum-free medium for 30 min at 37 ℃ for starvation. Subsequently, 70 kDa FITC-conjugated dextran (Cat. No. ST2947, Beyotime) at a final concentration of 0.5 mg mL^-1^ and equal amounts of freshly isolated mitochondria were simultaneously added to the culture medium, followed by 60 min of incubation to facilitate fluid-phase uptake. After incubation, the supernatant was discarded, and cells were thoroughly washed three times with PBS to remove uninternalized extracellular dextran. Cells were then harvested and resuspended for flow cytometric quantification of intracellular fluorescence intensity, which reflected the endocytic capacity of macrophages.

*Seahorse assay*: Cellular mitochondrial respiration and glycolytic capacities were quantified using a Seahorse XF extracellular flux analyzer. Briefly, BMDMs were seeded in XF culture plates at an appropriate density and cultured overnight. Prior to detection, the culture medium was replaced with pre-warmed XF assay medium, and BMDMs were incubated in a non-CO_2_ incubator at 37 ℃ for 60 min to stabilize extracellular pH. For mitochondrial stress tests, oligomycin, FCCP, and rotenone/antimycin A were sequentially injected to measure basal respiration and maximal respiration. For glycolysis stress tests, glucose, oligomycin, and 2‑Deoxy‑D‑glucose (2-DG) were added sequentially to evaluate basal glycolysis and glycolytic capacity. The real-time OCR and ECAR values were recorded and analyzed via Seahorse Wave software, with all data normalized to cell count. Calculation formulas are listed below: Glycolysis = (Maximum rate measurement before Oligomycin injection) - (Last rate measurement before Glucose injection); Glycolytic Capacity = (Maximum rate measurement after Oligomycin injection) - (Last rate measurement before Glucose injection); Basal Respiration = (Last rate measurement before first injection) - (Non-Mitochondrial Respiration Rate); Maximal Respiration = (Maximum rate measurement after FCCP injection) - (Non-Mitochondrial Respiration).

*Determination of cROS and mtROS*: BMDMs were isolated and cultured in six-well plates at a density of 1× 10^6^ cells per well and confocal dishes at a density of 1× 10^5^ cells per dish. The cells were then stimulated with TBHP (300 μM) for 2 h to simulate oxidative stress. After that, the cells were washed by PBS to wipe off TBHP and then cultured with MQ^gel^, isolated mitochondria (Mito), or both components (MQ^gel^@Mito) for 6 h, respectively. Then the cells were washed and incubated with DCFH-DA (Cat. No. S0033S, Beyotime) or MitoSOX Red (Cat. No. HY-D1055, MCE) solution at 37℃ for 20 min in the dark. The cells in six-well plates were washed for 3 times with PBS and harvested to conduct the flow cytometry by a flow cytometer (C6 Plus, BD Biosciences, San Jose, CA, USA). The results were analyzed by the Flowjo software v10.8.1. The cells seeded in confocal dishes were washed three times and stained with Hoechst 33342 (Cat. No. C1026, Beyotime) for 10 minutes. Then the cells were observed by the laser confocal microscopy (TCS SP5II, Leica, Ernst-Leitz-Strasse, Germany).

*Determination of apoptosis*: BMDMs were isolated and cultured in six-well plates at a density of 1× 10^6^ cells per well and treated with TBHP, MQ^gel^, mitochondria following the same procedure as described in the Supporting experimental methods section of *Determination of cROS and mtROS*. The apoptosis kit (Cat. No. C1062L, Beyotime) was used to assess the total apoptosis rate of cells stimulated by oxidative stress. After staining, the cells were harvested to conduct the flow cytometry using a flow cytometer (C6 Plus, BD Biosciences, San Jose, CA, USA). The results were analyzed by the Flowjo software v10.8.1. The total apoptosis rate was defined as the sum of the early apoptosis rate (Annexin V^+^/PI^-^) and the late apoptosis rate (Annexin V^+^/PI^+^).

*Histological staining*: On day 7 and day 28 post-MI, rats were euthanized and hearts were harvested and fixed with 4% PFA, embedded in paraffin, and sliced into sections with 4 μm-thick. After undergoing baking (60 ℃, 1 h), dewaxing, and rehydration, the slices were used for H&E staining (Cat. No. C0105S, Beyotime) and Masson’s trichrome staining (Cat. No. DC0032, Leagene) according to the standard protocols.

*The absorption of MQ^gel^@Mito in rat hearts*: MQ^gel^ was coupled with FITC and then assembled the MQ^gel^@Mito system. A total of 100 μL MQ^gel^@Mito was injected into the infarct border zone of rat hearts. Rats were sacrificed at different time points, and intact hearts were isolated subsequently. The cardiac fluorescence signals were detected using the Maestro imaging system (CRI, USA).

*The colocalization of donor mitochondria with macrophages or cardiomyocytes*: Freshly isolate donor mitochondria were labeled with MTR (Cat. No. C1032, Beyotime), and the labeled mitochondria were used to prepare MTR-labeled MQ^gel^@Mito composites. A total of 100 μL MTR-labeled donor mitochondria (Mito) or MQ^gel^@Mito were injected into the infarct border zone of rat hearts and the hearts were harvested at the scheduled time. After that, the hearts were preserved in the Tissue-Tek® O.C.T. Compound (Cat. No. 4583, Sakura Finetek) and then rapidly frozen in liquid nitrogen. The tissue was sliced into sections with 10 μm-thick. To investigate the colocalization of donor mitochondria with macrophages and cardiomyocytes, immunofluorescent staining was performed. The slices were incubated overnight at 4 ℃ with primary antibodies (CD68, Cat. No. ab283654, Abcam; cTnT, Cat. No. ab209813, Abcam), respectively. Immunofluorescent images were observed and captured using a laser confocal microscope (TCS SP5II, Leica, Ernst-Leitz-Strasse, Germany). The overlap of MTR and CD68 (or cTnT) confirmed the colocalization.

*WGA and TUNEL staining*: After undergoing baking, dewaxing, rehydration, and antigen retrieval, the slices were incubated with WGA staining solution (Cat. No. L4895, Sigma-Aldrich) for 30 min at 37 ℃. After three washes, the slices were mounted with an antifade mounting medium containing DAPI (Cat. No. ab104139, Abcam). For apoptosis assessment of cardiac tissue, the slices were stained with the TUNEL BrightRed Apoptosis Detection Kit (Cat. No. A113, Vazyme) according to the standard protocol. Fluorescent images of WGA and TUNEL staining were observed and captured using a laser confocal microscope (TCS SP5II, Leica, Ernst-Leitz-Strasse, Germany).

**Supporting figures**

**Fig. S1.** (A) Representative CD spectra of MOTS‑c, Q11, and MQ peptides. CD, circular dichroism. (B) Quantitative proportion of α‑helix and antiparallel β‑sheet in the MQ peptide.

**Fig. S2.** Representative TEM images showing the structures of MOTS‑c, Q11, and MQ peptides.

**Fig. S3.** Preparation process and representative images of the MQ^gel^.

**Fig. S4.** Rheological properties of MQ^gel^ at concentrations of 20, 30, and 40 mg mL^-1^ under frequency sweeps ranging from 0.1 to 34 rad s^-1^. G′ represents storage modulus; G′′ represents loss modulus.

**Fig. S5.** ATP production by mitochondria protected with Q11^gel^, MOTS-c, and MQ^gel^ (n = 3). Data are presented as mean ± SD; ns, not significant. **p* < 0.05, ****p* < 0.001.

**Fig. S6.** Storage moduli (G′) and loss moduli (G′′) of 30 mg mL^-1^ MQ^gel^ and MQ^gel^@Mito at a fixed frequency of 1 rad s^-1^ (n = 3).

**Fig. S7.** Self-healing properties of 30 mg mL^-1^ MQ^gel^ under alternating oscillatory strains: high strain (100%) and low strain (1%). G′ represents storage modulus; G′′ represents loss modulus.

**Fig. S8.** Representative SEM images of 30 mg mL^-1^ MQ^gel^.

**Fig. S9.** MitoSOX fluorescence intensity of isolated mitochondria under TBHP exposure (n = 3). Data are mean ± SD; ns, not significant. **p* < 0.05, ****p* < 0.001.

**Fig. S10.** (A) Cell viability of BMDMs treated with the MQ peptide (n = 3). (B) Representative live/dead fluorescence images of BMDMs treated with the MQ peptide. Data are mean ± SD. **p* < 0.05, ****p* < 0.001.

**Fig. S11.** Representative fluorescence images showing cellular uptake of FITC‑labeled MQ peptide by macrophages. F‑actin cytoskeleton was stained with phalloidin, and cell nuclei were stained with Hoechst.

**Fig. S12.** (A-B) Representative flow cytometry histograms (A) and corresponding MFI (B) of BMDMs receiving transplanted donor mitochondria stained with MTR after 12 h of mitochondria transplantation (n = 3). MTR, MitoTracker Red. Data are mean ± SD. ****p* < 0.001.

**Fig. S13.** Validation of *AMPKα1* knockdown efficiency in BMDMs. (A) Relative mRNA levels of *AMPKα1* and *AMPKα2* determined via qPCR (n = 3). (B) qPCR-based quantification of *AMPKα1* silencing efficiency at the mRNA level (n = 3). (C-D) Representative western blot images (C) and quantitative analysis (D) of AMPKα protein expression following si‑*AMPKα1* transfection (n = 3). Data are mean ± SD; ***p* < 0.01, ****p* < 0.001.

**Fig. S14.** *AMPKα1* is required for MQ-mediated enhancement of mitochondrial uptake in BMDMs detected by flow cytometry. (A-B) Representative flow cytometry histograms (A) and corresponding MFI quantification (B) for donor mitochondrial uptake in BMDMs after 12 h of mitochondrial transplantation. Cells were treated with naked mitochondria (Mito), MQ@Mito, or MQ@Mito+si-*AMPKα1* (n = 3). Data are mean ± SD; **p* < 0.05, ***p* < 0.01, ****p* < 0.001.

**Fig. S15.** *AMPKα1* is required for MQ-mediated enhancement of mitochondrial uptake in BMDMs detected by western blot. (A-B) Western blot images (A) and corresponding quantification of the p‑AMPKα/AMPKα ratio (B) in BMDMs treated with naked mitochondria (Mito), MQ@Mito, or MQ@Mito+si-*AMPKα1* (n = 3). Data are mean ± SD; ns, not significant; ****p* < 0.001.

**Fig. S16.** MQ promotes mitochondrial internalization via AMPK signaling activation in BMDMs. (A) Colocalization of MTG-labeled recipient mitochondria (green) and MTR-labeled donor mitochondria (red) in BMDMs after 2 and 12 h of mitochondrial transplantation. Cell nuclei (blue) were counterstained. MTG, MitoTracker Green; MTR, MitoTracker Red. (B-C) Representative western blot images (B) and corresponding quantification of the p‑AMPKα/AMPKα ratio (C) in BMDMs treated with naked mitochondria (Mito), MQ@Mito, or MQ@Mito+CC (n = 3). CC, Compound C. Data are presented as mean ± SD; **p* < 0.05, ***p* < 0.01.

**Fig. S17.** Dextran uptake assay showing that *AMPKα1* is required for MQ-mediated enhancement of macropinocytosis in BMDMs. (A, B) Representative flow cytometry histograms (A) and corresponding MFI quantification (B) for dextran uptake in BMDMs treated with naked mitochondria (Mito), MQ@Mito, or MQ@Mito+si‑*AMPKα1* (n = 3). Data are mean ± SD; ns, not significant; ***p* < 0.01, ****p* < 0.001.

**Fig. S18.** *AMPKα1* is required for MQ-mediated enhancement of mitochondrial uptake in LPS-pretreated M1-type BMDMs. (A-B) Representative flow cytometry histograms (A) and corresponding MFI quantification (B) of donor mitochondrial uptake in M1-type BMDMs treated with naked mitochondria (Mito), MQ@Mito, or MQ@Mito+si-*AMPKα1* (n = 3). Data are mean ± SD; **p* < 0.05, ***p* < 0.01.

**Fig. S19.** Representative immunofluorescence images showing CD86 (green) expression in LPS- stimulated BMDMs under different treatment conditions. F-actin (red) and cell nuclei (blue) were counterstained.

**Fig. S20.** Effects of MQ@Mito on LPS‑induced M1 macrophage polarization. (A-B) Representative pseudocolor density plots (A) and quantitative analysis (B) of F4/80^+^CD86^+^ M1 macrophage proportions when MQ@Mito was co‑administered with LPS stimulation (n = 3). (C-D) Representative pseudocolor density plots (C) and quantitative analysis (D) of F4/80^+^CD86^+^ M1 macrophage proportions when MQ@Mito was applied after LPS stimulation (n = 3). Data are presented as mean ± SD; ns, not significant; **p* < 0.05, ****p* < 0.001.

**Fig. S21.** (A) Cell viability of H9c2 cardiomyocytes treated with the MQ peptide (n = 3). (B) Representative live/dead fluorescence images of H9c2 cardiomyocytes treated with the MQ peptide. Data are mean ± SD. **p* < 0.05.

**Fig. S22.** Representative fluorescence images showing mitochondrial internalization in H9c2 cardiomyocytes. F-actin (green) and cell nuclei (blue) were counterstained.

**Fig. S23.** Cell viability of H9c2 cardiomyocytes under TBHP‑induced oxidative stress (n = 3). Data are presented as mean ± SD; ns, not significant; **p* < 0.05, ****p* < 0.001.

**Fig. S24.** Degradation of MQ^gel^@Mito in rat hearts. (A) Representative fluorescence images of harvested rat hearts following MQ^gel^@Mito injection at different time points (n = 3). (B) Fluorescence intensity of rat hearts post-injection (n = 3). Data are presented as mean ± SD.

**Fig. S25.** Quantitative echocardiographic analysis of cardiac function in rats. (A-D) Comparison of LVEF (A), LVFS (B), LVIDd (C), and LVIDs (D) on day 7 and day 28 post-MI (n = 5). (E-H) Quantification and comparison of LVEDV (E, F) and LVESV (G, H) on day 7 and day 28 post-MI (n = 5). Data are presented as mean ± SD; ns, not significant; **p* < 0.05, ***p* < 0.01, ****p* < 0.001.

**Fig. S26.** Histological H&E staining of major organs (liver, spleen, lung, and kidney) from rats receiving intramyocardial MQ^gel^@Mito injection versus the Sham group on day 7 and day 28 post-MI.

**Fig. S27.** Blood biochemical quantification of hepatic and renal function markers in different rat groups (n = 3). (A-B) Serum levels of hepatic function markers ALT and AST (n = 3). ALT, alanine transaminase; AST, aspartate transaminase. (C-D) Serum levels of renal function markers Cr and Urea (n = 3). Cr, creatinine. Data are mean ± SD; ns, not significant.

**Fig. S28.** TNF-α concentrations in the infarct border zone of rat hearts on day 7 post-MI (n = 3). Data are mean ± SD; **p* < 0.05, ***p* < 0.01.

**Fig. S29.** Representative TEM images of mitochondria in the infarct border zone of rat hearts on day 7 and day 28 post-MI.

**Fig. S30.** Assessment of mitochondrial function in the infarct border zone on day 28 post‑MI. (A) Activity of mitochondrial respiratory chain Complex I (n = 3). (B) Activity of mitochondrial respiratory chain Complex V (n = 3). (C) Total ATP content normalized per gram myocardial tissue (n = 3). Data are presented as mean ± SD; **p* < 0.05, ***p* < 0.01, ****p* < 0.001.
